# Clinical outcomes and quality of life after contemporary isolated coronary bypass grafting: a prospective cohort study

**DOI:** 10.1097/JS9.0000000000000259

**Published:** 2023-03-13

**Authors:** Sigrid Sandner, Martin Misfeld, Etem Caliskan, Andreas Böning, Jose Aramendi, Sacha P. Salzberg, Yeong-Hoon Choi, Louis P. Perrault, Ilker Tekin, Gregorio P. Cuerpo, Jose Lopez-Menendez, Luca P. Weltert, Johannes Böhm, Markus Krane, José M. González-Santos, Juan-Carlos Tellez, Tomas Holubec, Enrico Ferrari, Gheorghe Doros, Clara J. Vitarello, Maximilian Y. Emmert

**Affiliations:** a Medical University of Vienna, Vienna, Austria; b Royal Prince Alfred Hospital; c Institute of Academic Surgery at Royal Prince Alfred Hospital; d The Baird Institute of Applied Heart and Lung Surgical Research, Sydney; e Medical School, University of Sydney, Camperdown, New South Wales, Australia; f University Department of Cardiac Surgery, Leipzig Heart Center, Leipzig; g Charité Universitätsmedizin Berlin; h Deutsches Herzzentrum der Charité (DHZC), Department of Cardiothoracic and Vascular Surgery, Berlin; i Justus Liebig University Giessen; j Kerckhoff Heart Center Bad Nauheim, Campus Kerckhoff Justus-Liebig University Giessen, Giessen; k German Heart Center Munich, Munich; l University Hospital Frankfurt, Frankfurt, Germany; m Montreal Heart Institute, Montreal, Canada; n Manavgat Government Hospital, Manavgat; o Bahçeşehir University Faculty of Medicine, Istanbul, Turkey; p European Hospital, Rome, Italy; q Hospital General Universitario Gregorio Marañón; r Hospital Universitario Ramon y Cajal, Madrid; s Hospital Universitario Virgen Macarena, Seville; t Hospital de Cruces, Barakaldo; u Hospital Universitario de Salamanca, Salamanca, Spain; v Swiss Heart Clinic, Zurich; w Cardiocentro Ticino Institute, EOC, Lugano, Switzerland; x Yale University School of Medicine, New Haven, Connecticut; y Boston University; z Boston Clinical Research Institute (BCRI), Boston, Massachusetts, USA

**Keywords:** clinical outcomes, coronary artery bypass grafting, endothelial damage inhibitor, quality of life, saphenous vein graft

## Abstract

The objective of the European Multicenter Registry to Assess Outcomes in coronary artery bypass grafting (CABG) patients (DuraGraft Registry) was to determine clinical outcomes and quality of life (QoL) after contemporary CABG that included isolated CABG and combined CABG/valve procedures, using an endothelial damage inhibitor (DuraGraft) intraoperatively for conduit preservation. Here, we report outcomes in the patient cohort undergoing isolated CABG.

**Methods:**

The primary outcome was the composite of all-cause death, myocardial infarction (MI), or repeat revascularization (RR) [major adverse cardiac events (MACE)] at 1 year. Secondary outcomes included the composite of all-cause death, MI, RR, or stroke [major adverse cardiac and cerebrovascular events (MACCE)], and QoL. QoL was assessed with the EuroQol-5 Dimension questionnaire. Independent risk factors for MACE at 1 year were determined using Cox regression analysis.

**Results:**

A total of 2532 patients (mean age, 67.4±9.2 years; 82.5% male) underwent isolated CABG. The median EuroScore II was 1.4 [interquartile range (IQR), 0.9–2.3]. MACE and MACCE rates at 1 year were 6.6% and 7.8%, respectively. The rates of all-cause death, MI, RR, and stroke were 4.4, 2.0, 2.2, and 1.9%, respectively. The 30-day mortality rate was 2.3%. Age, extracardiac arteriopathy, left ventricular ejection fraction less than 50%, critical operative state, and left main disease were independent risk factors for MACE. QoL index values improved from 0.84 [IQR, 0.72–0.92] at baseline to 0.92 [IQR, 0.82–1.00] at 1 year (*P*<0.0001).

**Conclusion:**

Contemporary European patients undergoing isolated CABG have a low 1-year clinical event rate and an improved QoL.

## Graphical abstract

**Figure:**
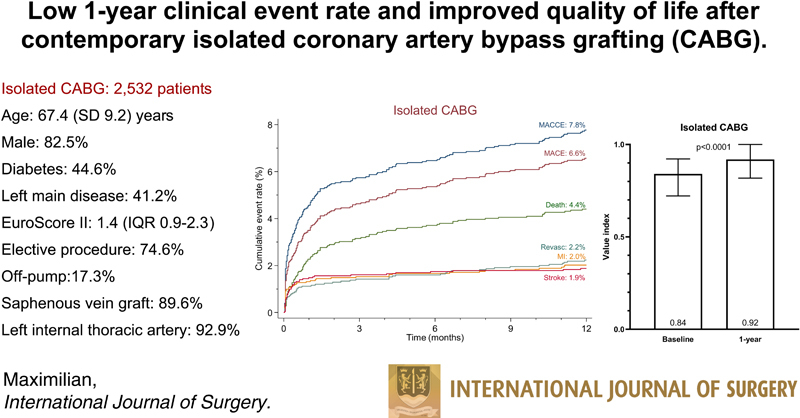

Highlights

- The 1-year clinical event rate is low in ‘all-comer’ isolated coronary artery bypass grafting patients.
- Quality of life was improved at 1 year.

## Introduction

Coronary artery bypass grafting (CABG) is the most frequently performed cardiac surgical procedure and the preferred revascularization strategy for patients with complex coronary artery disease^1^. However, data on contemporary outcomes after CABG in Europe are sparse, particularly reports including quality of life (QoL).

Saphenous vein grafts (SVGs) are the most commonly used conduit for CABG, yet their patency remains limited, thereby increasing the recurrence of symptoms of angina and rates of myocardial infarction (MI) and repeat revascularization (RR)^2^–^5^. SVG patency is mainly dependent on protection of the structural and functional integrity of the vascular endothelium during conduit harvest^6^–^8^. Recent studies have suggested a protective effect of DuraGraft, an endothelial damage inhibitor, for intraoperative SVG treatment^9^,^10^. Optimal storage conditions for free arterial grafts are still unclear^11^.

Clinical outcomes using endothelial damage inhibitors have not yet been reported in large series of patients. The European Multicenter Registry to Assess Outcomes in CABG Patients (DuraGraft Registry) assessed clinical outcomes and QoL in a contemporary cohort of European patients undergoing CABG with the treatment of vascular conduits using DuraGraft, including isolated CABG and a combined CABG/valve procedure. Here, we report clinical outcomes and QoL in the patient cohort undergoing isolated CABG.

## Patients and methods

### Study design and patient population

The full protocol and rationale for the European Multicenter Registry to Assess Outcomes in CABG Patients have been described previously^12^ and this work is reported in line with the Strengthening The Reporting Of Cohort Studies in Surgery (STROCSS) criteria^13^. Briefly, this prospective multicenter cohort study enrolled patients (male or female) at least 18 years of age undergoing CABG (isolated CABG or a combined CABG/valve procedure) with the use of at least 1 SVG and/or at least 1 free arterial graft, and use of DuraGraft. Patients participating in a medical device study or those who had received an investigational study drug in the month before enrollment were excluded. Patients referred for CABG at participating centers were screened for eligibility and approached to obtain informed consent. The primary outcome was the occurrence of major adverse cardiac events (MACE), defined as a composite of death, MI, or RR. A secondary outcome was the occurrence of major adverse cardiac and cerebrovascular events (MACCE), defined as the composite of death, MI, RR, or stroke. All components of the composite outcomes were also assessed individually. Definitions of events are provided in the Supplementary Materials. All outcome-related events were adjudicated per the event definitions by an independent event adjudication committee. An additional secondary outcome was QoL. QoL was assessed by the EuroQol-5 Dimension (EQ-5D) questionnaire at baseline and at 1 year after the procedure. The individual patient scores for the five dimensions of QoL assessed by the EQ-5D were converted into an index value using the value set for the Spanish population^14^. Index values range from 0 to 1, with higher values indicating a better QoL. The DuraGraft Registry will follow patients out to 5 years after CABG. Here, outcomes are reported at 30 days and 1 year.

### Intraoperative treatment of grafts

All CABG procedures were performed according to participating centers’ standard of care. Harvesting technique and grafting strategy were not mandated in the Registry protocol and were left to individual surgeons’ discretion. All SVG and free arterial grafts were intraoperatively flushed with and/or stored in the endothelial damage inhibitor DuraGraft (Marizyme, Jupiter, Florida, USA) as per the Registry protocol. DuraGraft is an ionically and pH-balanced physiological salt solution containing glutathione, l-ascorbic acid, and l-arginine as an antioxidant and generator of nitric oxide that has previously been shown to protect structure and function of the vascular endothelium, and mitigate ischemic and reperfusion damage during graft storage^8^,^10^,^15^–^17^.

### Statistical analysis

Continuous and categorical variables are reported as a mean and standard deviation (if normally distributed) or as the median and interquartile range [(IQR), if not normally distributed] or a frequency and percentage. The number of patients for whom data was available was used as the denominator. Data were generated for all enrolled patients and subgroups of patients undergoing isolated CABG and patients undergoing combined CABG and valve surgery. Survival curves for time-to-event variables were constructed on the basis of all available follow-up data using the Kaplan–Meier method. Multivariable Cox proportional hazards regression was performed to identify independent predictors of MACE and MACCE (two-sided 0.05 for significance). The multivariable model was built with variables selected for clinical interest. Multicollinearity of covariates was tested, and variables included in the final model were selected to avoid overfitting. The candidate variables for the multivariable models included age (incremental steps of 10 year), male gender, renal impairment (severe, moderate), extracardiac arteriopathy, poor mobility due to noncardiac reasons, previous cardiac surgery, pulmonary disease (chronic obstructive pulmonary disease/emphysema/asthma), critical preoperative status, insulin-dependent diabetes mellitus, New York Heart Association (NYHA) grade (classes II, III, and IV), Canadian Cardiovascular Society (CCS) grade (class IV), ejection fraction category (<50%), MI within 90 days, pulmonary hypertension, operative status (emergency, urgent), left main disease, category of graft used (only SVG and only arterial grafts), and use of cardiopulmonary bypass. All analyses were performed using SAS (version 9.4, Cary, North Carolina, USA).

## Results

### Patient baseline and procedural characteristics

A total of 2964 patients were enrolled between December 2016 and August 2019 at 45 centers in Austria, Germany, Ireland, Italy, Spain, Switzerland, Turkey, and the United Kingdom (Supplementary Materials, Supplemental Digital Content 1, http://links.lww.com/JS9/A83). An isolated CABG procedure was performed in 2532 patients (85.4%).

The mean age in the isolated CABG cohort was 67.4±9.2 years, and 82.5% of patients were male (Table 1). The prevalence of diabetes mellitus was 44.6%. Moderate to severe renal impairment was present in 53.6%, and 32.1% of patients had a reduced left ventricular ejection fraction less than 50%. The majority of patients (81.7%) presented with 3 vessel disease, and the left main disease was present in 41.2%. The median EuroScore II was 1.4 (IQR, 0.9–2.3). Procedures were mostly performed as elective surgeries (74.6%); off-pump CABG was used in 17.3% (Table 2). The open technique was the preferred method of SVG harvesting (80.6%). The SVG was the most frequently used conduit and was used in 89.6% of patients (Table 3). The majority of patients received either 1 (42.4%) or 2 (34.0%) SVG. A left internal thoracic artery was used in 92.9% of patients. Use of guideline-directed medical therapy was high, with 93.6% of patients receiving aspirin, 87.1% of patients receiving statins, and 85.4% of patients receiving β-blockers at discharge (Table S1, Supplemental Digital Content 1, http://links.lww.com/JS9/A83).

**Table 1:** Baseline characteristics of patients undergoing isolated coronary artery bypass grafting (CABG)

	Isolated CABG (*n*=2532)
Age, year, mean±SD	67.4±9.2 (2532)
Male	82.5 (2088/2532)
Caucasian ethnicity	87.9 (2219/2525)
BMI, kg/m^2^, mean±SD	28.5±4.5 (2532)
Smoking status	
Current smoker	19.7 (499/2530)
Ex-smoker	42.4 (1073/2530)
Never smoked	37.9 (958/2530)
Diabetes mellitus	44.6 (1130/2532)
Insulin-treated diabetes mellitus	14.5 (367/2532)
Hypertension	84.6 (2129/2516)
Dyslipidemia	77.0 (1925/2501)
Renal function^a^	
Normal	46.4 (1175/2532)
Moderately impaired	42.1 (1067/2532)
Severely impaired	11.5 (290/2532)
Cerebrovascular disease^b^	8.5 (214/2524)
Peripheral vascular disease	16.3 (409/2502)
Pulmonary disease	13.9 (352/2532)
Pulmonary hypertension	8.1 (206/2532)
Previous myocardial infarction	42.4 (1068/2517)
Anginal status CCS III and IV	32.8 (830/2532)
Atrial fibrillation/flutter	7.8 (197/2532)
Previous PCI	24.6 (623/2532)
Previous cardiac surgery	1.3 (32/2532)
Active endocarditis	0.0 (1/2532)
Extent of coronary artery disease	
1 vessel disease	1.9 (49/2517)
2 vessel disease	16.4 (412/2517)
3 vessel disease	81.7 (2056/2517)
Left main disease	41.2 (1033/2510)
LVEF≤50%	32.1 (814/2532)
EuroSCORE II, median (IQR, *n*)	1.4 (0.9–2.3, 2532)

a Severe renal impairment: creatinine clearance less than 50 ml/min; moderate renal impairment: creatinine clearance 50–85 ml/min.

b Previous stroke, transient ischemic attack, or coma.

CCS, Canadian Cardiovascular Society; IQR, interquartile range; LVEF, left ventricular ejection fraction; PCI, percutaneous coronary intervention.

**Table 2:** Procedural characteristics in patients undergoing isolated coronary artery bypass grafting (CABG)

	Isolated CABG (*n*=2532)
Status	
Elective	74.6 (1889/2532)
Urgent	24.1 (609/2532)
Emergent	1.3 (34/2532)
Salvage	0.0 (0/2532)
Off-pump	17.3 (437/2532)
Full sternotomy	99.7 (2517/2524)
Vein harvesting technique	
Open	80.6 (1904/2361)
Endoscopic	14.7 (348/2361)
Combined open and endoscopic	3.1 (73/2361)
No-touch	1.5 (36/2361)
Complete revascularization	81.0 (2049/2529)
Cumulative cross-clamp time (min)	69±26 (2075)
Cumulative bypass time (min)	100±35 (2088)

**Table 3:** Details of graft used in patients undergoing isolated coronary artery bypass grafting (CABG)

	Isolated CABG (*n*=2532)
Graft use per patient	
Saphenous vein	89.6 (2265/2528)
LITA	92.9 (2349/2528)
RITA	17.7 (447/2528)
Radial artery	11.3 (285/2528)
Total grafts per patient, mean±SD	2.7±0.8 (2528)
Vein grafts per patient, mean±SD	1.5±0.9 (2528)
Vein grafts per patient	
0	10.4 (263/2528)
1	42.4 (1072/2528)
2	34.0 (860/2528)
3	11.7 (296/2528)
≥4	1.5 (37/2528)
Arterial grafts per patient, mean±SD	1.2±0.6 (2528)
Arterial grafts per patient	
0	5.3 (135/2528)
1	70.5 (1781/2528)
≥2	24.2 (612/2528)
Distal anastomoses, mean±SD	3.0±0.9 (2507)
Myocardial territory grafted	
Left anterior descending	95.3 (2389/2507)
Circumflex artery	83.8 (2101/2507)
Right coronary artery	70.4 (1765/2507)

LITA, left internal thoracic artery; RITA, right internal thoracic artery.

Characteristics of the whole cohort (*n*=2964) and of patients who underwent a combined CABG/valve procedure (*n*=432, 14.6%) are presented in Tables S2–S5, Supplemental Digital Content 1, http://links.lww.com/JS9/A83 and S6–S9, Supplemental Digital Content 1, http://links.lww.com/JS9/A83, respectively.

### Clinical outcomes

**Figure 1:**
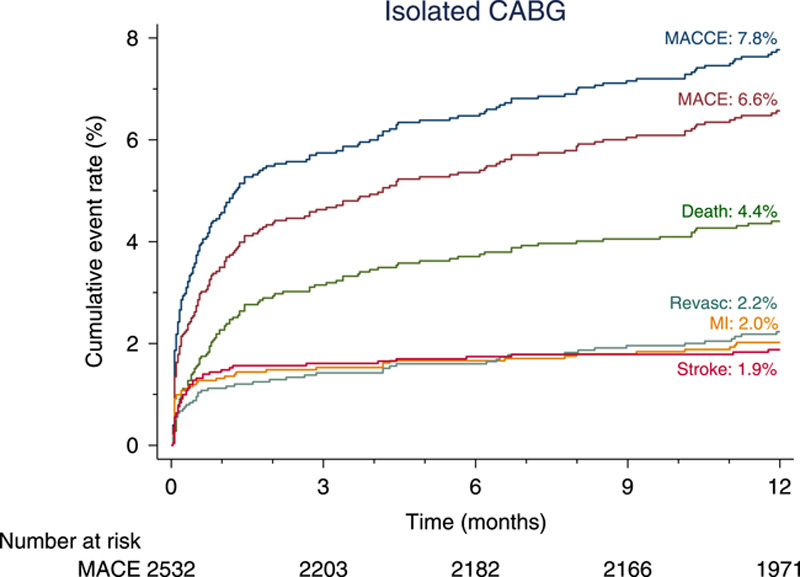
Cumulative incidence of the composite primary outcome (MACE) and secondary outcome (MACCE) and their individual components at 1 year. CABG, coronary artery bypass grafting; MACE, major adverse cardiac events; MACCE, major adverse cardiac and cerebrovascular events.

In patients undergoing isolated CABG, the rate of the primary composite endpoint of MACE was 6.6% at 1 year (Fig. 1, Table 4). The rates of the individual components of the primary endpoint were 4.4% for all-cause mortality, 2.0% for MI, and 2.2% for RR, respectively (Fig. 1). The rate of MACCE was 7.8% at 1 year, and the rate of stroke was 1.9%.

**Table 4:** Clinical outcomes after isolated coronary artery bypass grafting

	Isolated CABG (*n*=2532)	
	% (No. of events)	
	30 days	1 year
MACE	3.5 (88)	6.6 (160)
MACCE	4.6 (115)	7.8 (190)
All-cause death	2.3 (57)	4.4 (107)
Cardiovascular death	2.3 (57)	3.8 (92)
Myocardial infarction	1.3 (33)	2.0 (49)
All repeat revascularization	1.1 (28)	2.2 (53)
PCI	0.8 (21)	1.9 (45)
Re-CABG	0.3 (7)	0.3 (8)
Stroke	1.5 (37)	1.9 (46)

Percentages indicate cumulative event rates by Kaplan–Meier estimates.

CABG, coronary artery bypass grafting; MACE, major adverse cardiac events; MACCE, major adverse cardiac and cerebrovascular events; PCI, percutaneous coronary intervention.

**Figure 2:**
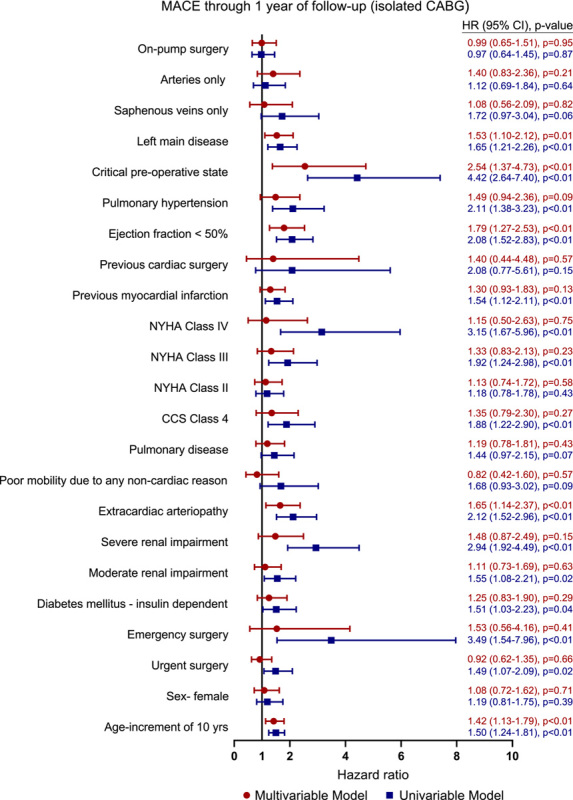
Hazard ratio for MACE in a univariable and multivariable model after isolated CABG. CABG, coronary artery bypass grafting; CCS, Canadian Cardiovascular Society; HR, hazard ratio; MACE, major adverse cardiac events; NYHA, New York Heart Association.

**Figure 3:**
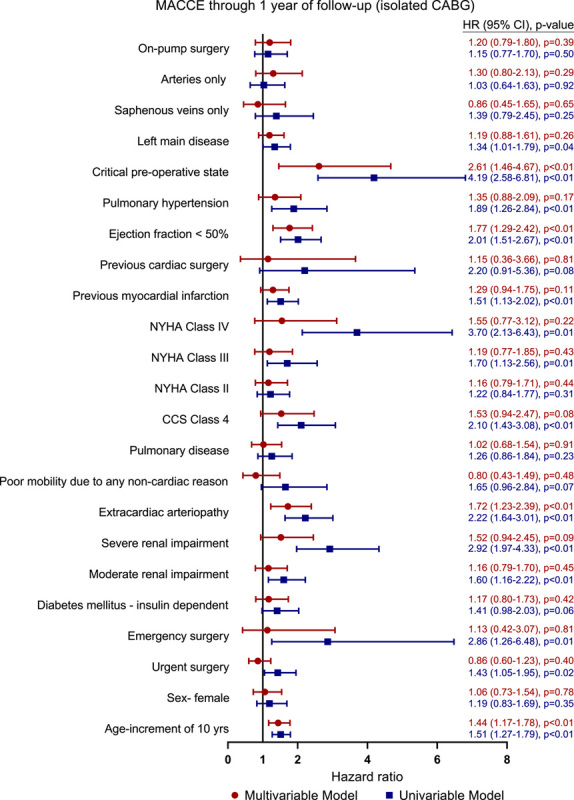
Hazard ratio for MACCE in a univariable and multivariable model after isolated CABG. CABG, coronary artery bypass grafting; CCS, Canadian Cardiovascular Society; HR, hazard ratio; MACCE, major adverse cardiac and cerebrovascular events; NYHA, New York Heart Association.

The rates of MACE and MACCE at 30 days were 3.5% and 4.6%, respectively (Table 4). Age (hazard ratio [HR], 1.42; 95% CI. 1.13–1.79; *P*<0.01), extracardiac arteriopathy (HR, 1.65; 95% CI, 1.14–2.37; *P*<0.01), left ventricular ejection fraction less than 50% (HR, 1.79; 95% CI, 1.27–2.53; *P*<0.001), critical preoperative status (HR, 2.54; 95% CI, 1.37–4.73; *P*<0.01), and left main disease (HR, 1.53; 95% CI, 1.10–2.12; *P*=0.01) were independent predictors of MACE at 1 year (Fig. 2 and Table S10, Supplemental Digital Content 1, http://links.lww.com/JS9/A83). Independent predictors of MACCE at 1 year were consistent with the predictors of MACE, with the exception of left main disease that did not reach statistical significance (Fig. 3 and Table S11, Supplemental Digital Content 1, http://links.lww.com/JS9/A83).

Clinical outcomes in the whole cohort and in patients who underwent a combined CABG/valve procedure are presented in Tables S12, Supplemental Digital Content 1, http://links.lww.com/JS9/A83-S17, Supplemental Digital Content 1, http://links.lww.com/JS9/A83 and Figures S1, Supplemental Digital Content 1, http://links.lww.com/JS9/A83 and S2, Supplemental Digital Content 1, http://links.lww.com/JS9/A83, respectively.

### QoL

**Figure 4:**
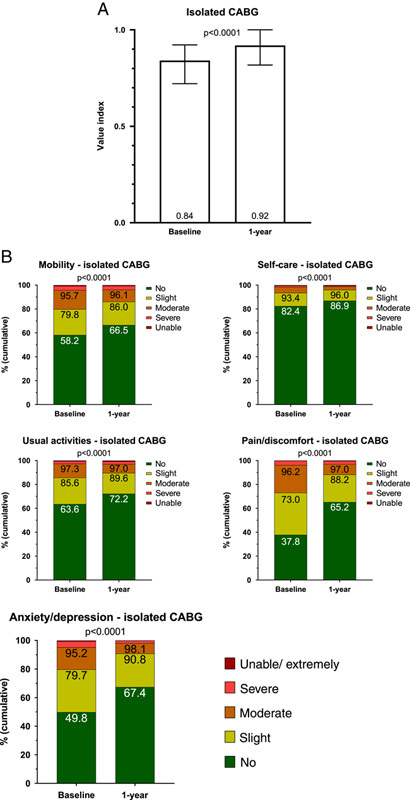
(A) Median and IQR of the EQ-5D index values at baseline and 1 year. (B) The five dimensions of the EQ-5D at baseline and 1 year. CABG, coronary artery bypass grafting; EQ-5D, EuroQol-5 Dimension; IQR, interquartile range.

In patients undergoing isolated CABG QoL by the EQ-5D index value significantly improved from baseline to 1 year: baseline: 0.84 [IQR, 0.72–0.92] and 1 year: 0.92 [IQR, 0.81–1.00], *P*< 0.0001 (Fig. 4A). Significant improvements occurred in all EQ-5D dimensions (Fig. 4B).

QoL by the EQ-5D index value in the whole cohort and in patients who underwent a combined CABG/valve procedure are presented in Figures S3–S6, Supplemental Digital Content 1, http://links.lww.com/JS9/A83, respectively.

## Discussion

The European Multicenter Registry to Assess Outcomes in CABG Patients (DuraGraft Registry) includes 2532 patients undergoing isolated CABG and provides the most comprehensive report to date on clinical outcomes and QoL after CABG surgery in a European all-comer population.

Our current understanding of contemporary outcomes of CABG is informed primarily by randomized clinical trials (RCTs) in highly selected patient populations with the well-known inherent limitation of generalizability. In contrast, the distinct advantage of large clinical registries is their ability to inform on the performance of surgical procedures in unselected, heterogeneous patient populations. The current Registry also bears the exceptional advantage that all clinical events were independently adjudicated, a measure otherwise limited to RCTs. However, large registry studies describing outcomes of contemporary CABG are sparse, and within Europe, are limited to national cohort analyses^18^,^19^. The Society of Thoracic Surgeons annually reports on operative outcomes of the most commonly performed cardiac surgical procedures in the United States but does not include longer-term outcome measures. In 2021, an operative mortality rate of 2.2% for isolated CABG procedures was reported^20^. Li *et al*.^21^ in a 2009 analysis of the Chinese national multicentre database that included 8120 isolated CABG patients, reported an in-hospital mortality of 2.2%. More recently, the E-CABG Registry was designed as an observational registry study including patients undergoing isolated CABG at six European centers; however, outcomes of the full cohort have not been reported yet^22^.

Despite increasing patient-related risk factors, operative outcomes for isolated CABG procedures have continually improved over the past two decades, likely due to improvements in operative technique and increased use of guideline-directed medical therapy^23^,^24^. The DuraGraft Registry isolated CABG cohort reflects the increasing prevalence of cardiovascular risk factors such as diabetes (44.6%), as well as comorbid conditions such as moderate to severe impairment of renal function (53.6%) and depressed left ventricular function (32.1%). Our study shows that the contemporary 1-year mortality rate for isolated CABG is 4.4%, with the majority of deaths adjudicated to cardiovascular causes. More than half of all deaths occur during the perioperative period, with a 30-day mortality rate of 2.3%. The rates of MACE and MACCE at 1 year are 6.6% and 7.8%, respectively. Known risk factors such as depressed left ventricular function, critical preoperative status and extracardiac arteriopathy, and notably, the presence of left main disease^25^ significantly predict MACE at 1 year. While the rate of MACCE at 1 year in the Synergy between TAXUS and Cardiac Surgery (SYNTAX) trial^26^ (recruitment 2005–2007) was 12.4% at 1 year, the more recent Ticagrelor in CABG (TiCAB) trial^27^ (recruitment 2013–2017) reported a rate of the composite of cardiovascular death, stroke, MI, or RR of 8.2% in the aspirin control group at 1 year. Most recently, the Fractional Flow Reserve versus Angiography for Multivessel Evaluation (FAME) 3 trial^28^ reported a MACCE rate of 6.9% in the CABG arm at 1 year, confirming the improvement of outcomes of CABG over the past decade, and the overall slightly lower event rates in the more selected patient populations of RCTs.

Although patient-reported health outcomes are increasingly frequently included as primary or secondary study endpoints in RCTs, they are rarely captured in registry studies^29^. In prespecified analyses of the Surgical Treatment for Ischemic Heart Failure (STICH) and SYNTAX trials, respectively, CABG was shown to improve QoL compared with medical therapy^30^ and percutaneous coronary intervention^31^. Our study conclusively shows that in an all-comer isolated CABG population health-related QoL as assessed by the EQ-5D questionnaire significantly increased from baseline to 1 year after CABG, most notably in the dimension pain and discomfort, with 65% of patients reporting no chest pain at 1 year. A similarly striking improvement was noted in the dimension of anxiety and depression, with 67% of patients reporting no symptoms at 1 year. This is of particular importance, as depression in patients after CABG surgery is associated with an increased risk of adverse cardiac events and hospital readmission^32^,^33^.

SVGs are the most commonly used conduits in CABG when grafting nonleft anterior descending coronary artery territories and were used in ∼90% of patients in the Registry. Future research must therefore be directed at optimizing SVG patency for CABG. All SVG and free arterial grafts were flushed with and/or stored in the endothelial damage inhibitor DuraGraft after harvesting^12^. DuraGraft has been shown to prevent ischemic reperfusion injury damage and to preserve the functionality and integrity of the endothelial cell structure in preclinical and clinical studies^15^–^17^. In a recent within-patient RCT assessing morphological changes of SVG by computed tomography, angiography DuraGraft had a favorable effect on early anatomical markers of SVG disease, such as SVG wall thickness at 12 months, particularly in the proximal segment of the graft^10^. In addition, a recent study showed that DuraGraft preserved the functionality and integrity of endothelial and intimal cells of radial artery grafts^15^. The majority of studies support an association of graft patency with clinical outcomes^34^, and the role of endothelial damage inhibitors on long-term clinical outcomes remains to be elucidated with longer follow-up of patients in the Registry.

### Limitations

The main limitation of this study is the nonrandomized design, precluding absolute inferences about the efficacy of DuraGraft in preventing cardiac events. However, considering the number of CABG procedures performed annually worldwide with frequent use of SVG, the unsuitability of conventional graft storage solutions to preserve SVG endothelial integrity and functionality, and the morbidity and healthcare costs associated with SVG failure, our study addresses a timely and important medical question. DuraGraft is currently not routinely used for CABG; however, no guideline recommendations exist regarding the use of solutions for intraoperative graft preservation and storage. Another limitation of the current study is that cardiac biomarkers were not systematically collected periprocedurally, which may potentially result in an underreporting of the MI rate. Graft patency was not systematically assessed, and complete revascularization was not defined in the protocol. In addition, the harvesting technique and grafting strategy were not mandated in the protocol. Finally, it must be noted that the direct comparison of outcome rates between this registry and other studies may be limited by the heterogeneity of outcome definitions used in the respective studies.

## Conclusions

The European Multicenter Registry to Assess Outcomes in CABG Patients provides valuable longitudinal data on contemporary CABG practice in a European ‘all-comers’ population and shows that QoL improves significantly over 1 year after CABG. Randomized clinical studies are warranted to evaluate the effect of endothelial damage inhibitors on graft patency and clinical outcomes.

## Ethics approval

The study complied with the provisions of the Declaration of Helsinki. The Ethical Committee at each study center approved the study protocol. Ethical Committee approval was obtained for the first participating site on 28 September 2016 in Vitoria, Spain (No.: PI2016118) and consecutively at all new participating sites. All patients provided written informed consent.

## Registration

The European Multicenter Registry to Assess Outcomes in CABG Patients is registered at clinicaltrials.gov (NCT02922088) and can be accessed here: https://clinicaltrials.gov/ct2/show/NCT02922088.

## Sources of funding

This work was supported by Marizyme, Jupiter, Florida, USA.

## Author contribution

The first author S.S. and the corresponding author M.Y.E. were involved with data collection, analysis, and writing of the paper. All authors read, critically revised, and approved the final manuscript prior to submission.

## Conflicts on interest disclosure

E.C., S.S., M.M., J.A., S.P.S., Y.H.C., and A.B. are members of the Registry Advisory Committee (RAC). L.P.P. is a member of the RAC and is a consultant for Marizyme. MYE is the principal investigator of the registry, the chair of the RAC and a consultant for Marizyme. E.F. received research grants from Somahlution, a Marizyme company. Other authors have nothing to disclose.

## Research registration unique identifying number (UIN)

1. Name of the registry: The European Multicenter Registry to Assess Outcomes in CABG Patients is registered at clinicaltrials.gov
2. Unique identifying number or registration ID: NCT02922088
3. Hyperlink to your specific registration (must be publicly accessible and will be checked): https://clinicaltrials.gov/ct2/show/NCT02922088

## Guarantor

All accept full responsibility for the work and/or the conduct of the study. They had access to the data and controlled the decision to publish. All authors read, critically revised, and approved the final manuscript prior to submission.

## Data availability statement

Data are held by the study sponsor and are available upon reasonable request.

## Appendix 1. Members of Infection Control Teams participating in the program

Daniel Zimpfer, Stefanie Stasek, Medical University Vienna; Ulvi Cenk Oezpeker, Michael Grimm, Medical University of Innsbruck; Bernhard Winkler, Martin Grabenwöger, Departement Hospital Clinic Floridsdorf/ Vienna Heart Center; Michaele Andrä, Klinikum Klagenfurt am Wörthersee; Anas Aboud, Stephan Ensminger, University Hospital Schleswig-Holstein, Campus Lübeck; Michael A. Borger, University Department of Cardiac Surgery, Leipzig Heart Center, Leipzig, Germany; Bernd Niemann, Universitätsklinikum Gießen und Marburg GmbH; Arnaud Van Linden, University Hospital Frankfurt; Matthias Thielmann, Daniel Wendt, West-German Heart and Vascular Center, University Hospital Essen, University Duisburg-Essen; Assad Haneya, Katharina Huenges, Universitätsklinikum Schleswig-Holstein Campus Kiel; Herko Grubitzsch, Charité - Universitätsmedizin Berlin, corporate member of Freie Universität Berlin and Humboldt-Universität zu Berlin; Farhad Bakthiary, Universitätsklinikum Wuppertal; Jörg Kempfert, Adam J. Penkalla, Deutsches Herzzentrum Berlin; Bernhard C. Danner, Fawad A. Jebran, University Medical Center Göttingen; Carina Benstoem, Andreas Goetzenich, Christian Stoppe, RWTH Aachen University; Elmar W. Kuhn, Oliver J. Liakopoulos, University of Cologne; Stefan Brose, Klaus Matschke, Heart Center Dresden; Dave Veerasingam, University Hospital Galway; Kishore Doddakula, Cork University Hospital; Lorenzo Guerrieri Wolf, European Hospital, Rome; Giuseppe Filiberto Serraino, Pasquale Mastroroberto, Magna Graecia University of Catanzaro; Nicola Lamascese, Massimo Sella, ULSS 8 Berica, Vicenza; Edmundo R. Fajardo-Rodriguez, Hospital Universitario Ramon y Cajal, Madrid; Alejandro Crespo, Cruces University Hospital, Barakaldo Bizkaia; Angel L Fernandez Gonález, Hospital Universitario Santiago de Compostela; Alvaro Pedraz, Hospital General Universitario Gregorio Marañón, Madrid; Elena Arnáiz-García, Hospital Universitario de Salamanca; Ignacio Muñoz Carvajal, Hospital Universitario Reina Sofia, Córdoba; Adrian J. Fontaine, Hospital Universitario Puerta del Mar, Cadiz; José Ramón González, Complejo Hospitalario Universitario de Badajoz; Paloma Martinez, Complejo hospitalario Ruber Juan Bravo, Madrid; Jose Antonio Blazquez, Hospital Universitario La Paz, Madrid; Bella Ramirez, Hospital Universitario Virgen Macarena, Seville; Alejandro Adsuar-Gomez, Jose M. Borrego-Dominguez, Virgen del Rocio University Hospital, Seville; Christian Muñoz-Guijosa, Sara Badía-Gamarra, Hospital Universitario Germans Trias y Pujol, Barcelona; Rafael Sádaba, Alicia Gainza, Complejo Hospitalario de Navarra/Navarra Biomed, Pamplona; Manuel Castellá, Clinic Hospital, University of Barcelona; Gregorio Laguna, Javier A. Gualis, Hospital Universitario de León; Stefanos Demertzis, Cardiocentro Ticino Institute, Lugano; Jürg Grünenfelder, Swiss Heart Clinic AG, Zurich; Robert Bauernschmitt, University Hospital of Zurich; Amal K. Bose, Blackpool Teaching Hospitals; Nawwar Al-Attar, George Gradinariu, Golden Jubilee National Hospital, Glasgow

## Supplementary Material
