# Supplementary material for: Clinical outcomes and quality of life after contemporary isolated coronary bypass grafting: a prospective cohort study
Source: Int J Surg. 2023 Mar 13;109(4):707–15. doi: 10.1097/JS9.0000000000000259 (PMC10389413; doi:10.1097/JS9.0000000000000259)

**SUPPLEMENTARY MATERIALS**

**CLINICAL OUTCOMES AND QUALITY OF LIFE AFTER CONTEMPORARY ISOLATED CORONARY BYPASS GRAFTING –**

**A PROSPECTIVE COHORT STUDY**

**The European Multicenter Registry to Assess Outcomes in CABG Patients (DuraGraft Registry)**

**Table of Contents**

Registry Advisory Committee

Statistical Analysis

Clinical Event Adjudication Committee

European DuraGraft Registry Investigators

**Supplementary Methods**

Major adverse event definitions

Other definitions

**Supplementary Tables**

**Table S1.** Use of guideline-directed medical therapy for isolated CABG surgery patients

**Table S2.** Patient characteristics of the whole cohort including isolated CABG (n=2532) and combined CABG and valve surgery (n=432) patients

**Table S3.** Characteristics of the surgical procedures of the whole cohort including isolated CABG (n=2532) and combined CABG and valve surgery (n=432) patients

**Table S4.** Details of graft use of the whole cohort including isolated CABG (n=2532) and combined CABG and valve surgery (n=432) patients

**Table S5.** Use of guideline-directed medical therapy of the whole cohort including isolated CABG (n=2532) and combined CABG and valve surgery (n=432) patients

**Table S6.** Patient characteristics of patients who underwent combined CABG and valve surgery

**Table S7.** Characteristics of the surgical procedure of patients who underwent combined CABG and valve surgery

**Table S8.** Details of graft use of patients who underwent combined CABG and valve surgery

**Table S9.** Use of guideline-directed medical therapy of patients who underwent combined CABG and valve surgery

**Table S10.** Hazard ratio for a major adverse cardiac event in an univariable and multivariable model after isolated CABG

**Table S11.** Hazard ratio for a major adverse cardiac and cerebral event for isolated CABG patients in an univariable and multivariable model (n=2532)

**Table S12.** Clinical outcomes of the whole cohort including isolated CABG (n=2532) and combined CABG and valve surgery (n=432) patients

**Table S13.** Hazard ratio for a major adverse cardiac event for the whole cohort including isolated CABG (n=2532) and combined CABG and valve surgery (n=432) patients in an univariable and multivariable model

**Table S14.** Hazard ratio for a major adverse cardiac and cerebral event for the whole cohort including isolated CABG (n=2532) and combined CABG and valve surgery (n=432) patients in an univariable and multivariable model

**Table S15.** Clinical outcomes of combined CABG and valve surgery patients (n=432)

**Table S16.** Hazard ratio for a major adverse cardiac event for patients with combined CABG and valve surgery (n=432) in an univariable and multivariable model

**Table S17.** Hazard ratio for a major adverse cardiac and cerebral cardiac event for patients with combined CABG and valve surgery (n=432) in an univariable and multivariable model

**Supplementary Figures**

**Figure S1.** Cumulative incidence of adverse events for the whole cohort including isolated CABG (n=2532) and combined CABG and valve surgery (n=432) patients at 1 year

**Figure S2.** Cumulative incidence of adverse events after combined CABG and valve surgery at 1 year

**Figure S3.** Median and IQR of the EQ-5D index values for the whole cohort including isolated CABG (n=2532) and combined CABG and valve surgery (n=432) patients at baseline and 1 year

**Figure S4.** The five dimensions of the EQ-5D for the whole cohort including isolated CABG (n=2532) and combined CABG and valve surgery (n=432) patients at baseline and 1 year

**Figure S5.** Median and IQR of the EQ-5D index values for the combined CABG and valve patients (n=432) at baseline and 1 year

**Figure S6.** The five dimensions of the EQ-5D for the combined CABG and valve patients (n=432) at baseline and 1 year

# Registry Advisory Committee

| Maximilian Y Emmert (Chair) | Department of Cardiovascular Surgery, Charité Universitätsmedizin Berlin, Berlin, Germany  Department of Cardiothoracic and Vascular Surgery, German Heart Center Berlin, Berlin, Germany |
| --- | --- |
| Jose I. Aramendi | Division of Cardiac Surgery, Hospital de Cruces, Barakaldo, Spain |
| Andreas Böning | Universitätsklinikum Gießen und Marburg GmbH, Gießen, Germany |
| Etem Caliskan | Department of Cardiovascular Surgery, Charité Universitätsmedizin Berlin, Berlin, Germany  Department of Cardiothoracic and Vascular Surgery, German Heart Center Berlin, Berlin, Germany |
| Yeong-Hoon Choi | Kerckhoff Heart Center Bad Nauheim, Campus Kerckhoff Justus-Liebig University Giessen |
| Martin Misfeld | University Department of Cardiac Surgery, Leipzig Heart Center, Leipzig, Germany  Department of Cardiothoracic Surgery, Royal Prince Alfred Hospital, Sydney, Australia  Institute of Academic Surgery at RPA, Sydney, Australia  The Baird Institute of Applied Heart and Lung Surgical Research, Sydney, Australia  Medical School, University of Sydney, Australia |
| Louis P. Perrault | Montreal Heart Institute, Montreal, Canada |
| Sacha Salzberg | Swiss Heart Clinic, Zurich, Switzerland |
| Sigrid Sandner | Department of Cardiac Surgery, Medical University of Vienna, Vienna, Austria |

# Statistical Analysis

| Gheorghe Doros | Boston University, Boston, MA, USA |
| --- | --- |
| Clara J. Vitarello | Boston Clinical Research Institute (BCRI), Boston, MA, USA |
| C. Michael Gibson | Boston Clinical Research Institute (BCRI), Boston, MA, USA |

# Clinical Event Adjudication Committee

| Pascal Vranckx | Department of Cardiology and Critical Care Medicine, Hartcentrum Hasselt, Jessa Ziekenhuis, Belgium  Faculty of Medicine and Life Sciences, Hasselt University, Belgium |
| --- | --- |
| Jacques J Koolen | Catharina Hospital, Eindhoven, the Netherlands |

#

# European DuraGraft Registry Investigators

| **Country/site** | **Investigators** | **N⁰ of patients enrolled** |  |
| --- | --- | --- | --- |
| **AUSTRIA** |  | **269** |  |
| Medical University Vienna | Sigrid Sandner | 225 |  |
|  | Daniel Zimpfer |  |  |
|  | Stefanie Stasek |  |  |
| Medical University of Innsbruck | Ulvi Cenk Oezpeker | 10 |  |
|  | Michael Grimm |  |  |
| Departement Hospital Clinic Floridsdorf/ Vienna Heart Center | Bernhard Winkler | 14 |  |
|  | Martin Grabenwöger |  |  |
| Klinikum Klagenfurt am Wörthersee | Michaele Andrä | 20 |  |
| **GERMANY** |  | **985** |  |
| University Hospital Schleswig-Holstein, Campus Lübeck | Anas Aboud | 20 |  |
|  | Stephan Ensminger |  |  |
| University Department of Cardiac Surgery, Leipzig Heart Center, Leipzig, Germany | Martin Misfeld | 248 |  |
|  | Michael A. Borger |  |  |
| Universitätsklinikum Gießen und Marburg GmbH | Andreas Böning | 160 |  |
|  | Bernd Niemann |  |  |
| University Hospital Frankfurt | Tomas Holubec | 81 |  |
|  | Arnaud Van Linden |  |  |
| West-German Heart and Vascular Center, University Hospital Essen, University Duisburg-Essen | Matthias Thielmann | 55 |  |
|  | Daniel Wendt |  |  |
| Universitätsklinikum Schleswig-Holstein Campus Kiel | Assad Haneya | 7 |  |
|  | Katharina Huenges |  |  |
| German Heart Center Munich | Johannes Böhm | 115 |  |
|  | Markus Krane |  |  |
| Charité - Universitätsmedizin Berlin, corporate member of Freie Universität Berlin and Humboldt-Universität zu Berlin | Etem Caliskan | 182 |  |
|  | Herko Grubitzsch |  |  |
| Universitätsklinikum Wuppertal | Farhad Bakthiary | 7 |  |
| Deutsches Herzzentrum Berlin | Jörg Kempfert | 32 |  |
|  | Adam J. Penkalla |  |  |
| University Medical Center Göttingen | Bernhard C. Danner | 29 |  |
|  | Fawad A. Jebran |  |  |
| RWTH Aachen University | Carina Benstoem | 12 |  |
|  | Andreas Goetzenich |  |  |
|  | Christian Stoppe |  |  |
| University of Cologne | Elmar W. Kuhn | 5 |  |
|  | Yeong-Hoon Choi |  |  |
|  | Oliver J. Liakopoulos |  |  |
| Heart Center Dresden | Stefan Brose | 32 |  |
|  | Klaus Matschke |  |  |
| **IRELAND** |  | **31** |  |
| University Hospital Galway | Dave Veerasingam | 27 |  |
| Cork University Hospital | Kishore Doddakula | 4 |  |
| **ITALY** |  | **176** |  |
| European Hospital, Rome | Luca P. Weltert | 118 |  |
|  | Lorenzo Guerrieri Wolf |  |  |
| Magna Graecia University of Catanzaro | Giuseppe Filiberto Serraino | 48 |  |
|  | Pasquale Mastroroberto |  |  |
| ULSS 8 Berica, Vicenza | Nicola Lamascese | 10 |  |
|  | Massimo Sella |  |  |
| **SPAIN** |  | **1081** |  |
| Hospital Universitario Ramon y Cajal, Madrid | Jose Lopez-Menendez | 125 |  |
|  | Edmundo R. Fajardo-Rodriguez |  |  |
| Cruces University Hospital, Barakaldo Bizkaia | Jose I. Aramendi | 50 |  |
|  | Alejandro Crespo |  |  |
| Hospital Universitario Santiago de Compostela | Angel L Fernandez Gonález | 84 |  |
| Hospital General Universitario Gregorio Marañón, Madrid | Gregorio P. Cuerpo | 125 |  |
|  | Alvaro Pedraz |  |  |
| Hospital Universitario de Salamanca | José M. González-Santos | 100 |  |
|  | Elena Arnáiz-García |  |  |
| Hospital Universitario Reina Sofia, Córdoba | Ignacio Muñoz Carvajal | 56 |  |
| Hospital Universitario Puerta del Mar, Cadiz | Adrian J. Fontaine | 63 |  |
| Complejo Hospitalario Universitario de Badajoz | José Ramón González | 192 |  |
| Complejo hospitalario Ruber Juan Bravo, Madrid | Paloma Martinez | 25 |  |
| Hospital Universitario La Paz, Madrid | Jose Antonio Blazquez | 2 |  |
| Hospital Universitario Virgen Macarena, Seville | Juan-Carlos Tellez | 94 |  |
|  | Bella Ramirez |  |  |
| Virgen del Rocio University Hospital, Seville | Alejandro Adsuar-Gomez | 61 |  |
|  | Jose M. Borrego-Dominguez |  |  |
| Hospital Universitario Germans Trias y Pujol, Barcelona | Christian Muñoz-Guijosa | 40 |  |
|  | Sara Badía-Gamarra |  |  |
| Complejo Hospitalario de Navarra/Navarra Biomed, Pamplona | Rafael Sádaba | 13 |  |
|  | Alicia Gainza |  |  |
| Clinic Hospital, University of Barcelona | Manuel Castellá | 28 |  |
| Hospital Universitario de León | Gregorio Laguna | 23 |  |
|  | Javier A. Gualis |  |  |
| **SWITZERLAND** |  | **133** |  |
| Cardiocentro Ticino Institute, Lugano | Enrico Ferrari | 80 |  |
|  | Stefanos Demertzis |  |  |
| Swiss Heart Clinic AG, Zurich | Sacha Salzberg | 4 |  |
|  | Jürg Grünenfelder |  |  |
| University Hospital of Zurich | Robert Bauernschmitt | 49 |  |
| **TURKEY** |  | **160** |  |
| Manavgat Government Hospital | Ilker Tekin | 160 |  |
| **UNITED KINGDOM** |  | **126** |  |
| Blackpool Teaching Hospitals | Amal K. Bose | 60 |  |
| Golden Jubilee National Hospital, Glasgow | Nawwar Al-Attar | 66 |  |
|  | George Gradinariu |  |  |

# Supplementary Methods

## Major adverse event definitions

ALL-CAUSE MORTALITY

Cardiovascular mortality:

Any of the following criteria:

- Death due to proximate cardiac cause (e.g. myocardial infarction, cardiac tamponade, worsening heart failure)
- Death caused by non-coronary vascular conditions such as neurological events, pulmonary embolism, ruptured aortic aneurysm, dissecting aneurysm, or other vascular disease
- All procedure-related deaths, including those related to a complication of the procedure or treatment for a complication of the procedure
- All valve-related deaths including structural or non-structural valve dysfunction or other valve-related adverse events
- Sudden or unwitnessed death
- Arrythmia or cardiac arrest
- Death of unknown cause

Non-cardiovascular mortality:

Any death in which the primary cause of death is clearly related to another condition (e.g. trauma, cancer, suicide).

MYOCARDIAL INFARCTION (MI)

Evidence of myocardial injury with electrocardiographic changes and/or elevation of cardiac biomarkers.

REPEAT REVASCULARIZATION

Any ischemia- or symptom-driven percutaneous or surgical revascularization procedure performed involving the native coronary arteries or coronary artery bypass grafts after the index procedure.

STROKE

A focal neurological deficit of central origin lasting >24 h with or without confirmation with neuroimaging; or 2) a deficit lasting >72 h without the need for confirmation with neuroimaging

## Other definitions

CRITICAL OPERATIVE STATE

ventricular tachycardia or ventricular fibrillation or aborted sudden death, preoperative cardiac massage, preoperative ventilation before anaesthetic room, preoperative inotropes or IABP, preoperative acute renal failure (anuria or oliguria <10ml/hr).

EXTRACARDIAC ARTERIOPATHY

One or more of the following: claudication, carotid occlusion or >50% stenosis, amputation for arterial disease or previous or planned intervention on the abdominal aorta, limb arteries or carotids.

CHRONIC LUNG DISEASE: chronic obstructive pulmonary disease (COPD), emphysema and asthma with long term use of bronchodilators or steroids for lung disease

# Supplementary Tables

## Table S1. Use of guideline-directed medical therapy for isolated CABG surgery patients

|  | **Isolated CABG**  **(n=2532)** | |
| --- | --- | --- |
|  | Discharge | 1 Year |
| Aspirin | 93.6 (2363/2525) | 87.0 (1757/2020) |
| Other anti-platelet agent | 35.4 (894/2525) | 24.8 (500/2020) |
| Any anti-platelet agent | 97.0 (2449/2525) | 93.2 (1883/2020) |
| Vitamin K antagonist | 4.4 (110/2524) | 1.5 (30/2020) |
| DOAC | 4.8 (120/2524) | 6.6 (136/2067) |
| Statin | 87.1 (2199/2524) | 88.6 (1790/2021) |
| Other lipid-lowering agent (non-statin) | 3.7 (93/2524) | 9.4 (190/2021) |
| Beta-blocker | 85.4 (2155/2524) | 86.2 (1743/2021) |
| RAAS-active agent | 63.9 (1614/2524) | 63.8 (1289/2021) |

DOAC: direct oral anticoagulant; RAAS: renin-angiotensin-aldosterone-system.

## Table S2. Patient characteristics of the whole cohort including isolated CABG (n=2532) and combined CABG and valve surgery (n=432) patients

|  | **All patients**  **(n=2964)** |
| --- | --- |
| Age, y, mean ± SD | 67.8 ± 9.2 (2964) |
| Male | 82.3 (2439/2964) |
| Caucasian ethnicity | 88.3 (2610/2956) |
| BMI, kg/m^2^, mean ± SD | 28.4 ± 4.4 (2964) |
| Smoking status |  |
| Current smoker | 19.2 (568/2961) |
| Ex-smoker | 41.5 (1230/2961) |
| Never smoked | 39.3 (1163/2961) |
| Diabetes mellitus | 43.7 (1294/2962) |
| Insulin treated diabetes mellitus | 14.1 (418/2962) |
| Hypertension | 84.4 (2486/2946) |
| Dyslipidemia | 76.9 (2251/2929) |
| Renal function ^1^ |  |
| Normal | 44.5 (1318/2964) |
| Moderately impaired | 43.0 (1276/2964) |
| Severely impaired | 12.5 (370/2964) |
| Cerebrovascular disease ^2^ | 8.8 (259/2954) |
| Peripheral vascular disease | 16.0 (469/2927) |
| Pulmonary disease | 13.9 (412/2964) |
| Pulmonary Hypertension | 11.0 (326/2964) |
| Previous myocardial infarction | 40.1 (1182/2946) |
| Anginal status CCS III and IV | 31.2 (924/2964) |
| Atrial fibrillation/ flutter | 8.5 (251/2964) |
| Previous PCI | 24.0 (710/2964) |
| Previous cardiac surgery | 1.3 (38/2964) |
| Active endocarditis | 0.2 (5/2964) |
| Extent of coronary artery disease |  |
| 1 vessel disease | 4.5 (132/2945) |
| 2 vessel disease | 18.0 (530/2945) |
| 3 vessel disease | 77.5 (2283/2945) |
| Left main disease | 39.1 (1148/2934) |
| LVEF ≤ 50% | 32.5 (963/2964) |
| EuroSCORE II, median (IQR, n) | 1.6 (1.0-2.7, 2964) |

^1^ Severe renal impairment: creatine clearance < 50 ml/min; moderate renal impairment: creatine clearance 50-85 ml/min.

^2^ Previous stroke, transient ischemic attack or coma

BMI: body mass index; CCS: Canadian Cardiovascular Society; LVEF: left ventricular ejection fraction; PCI: percutaneous coronary intervention.

## Table S3. Characteristics of the surgical procedures of the whole cohort including isolated CABG (n=2532) and combined CABG and valve surgery (n=432) patients

|  | **All patients (n=2964)** |
| --- | --- |
| Status |  |
| Elective | 76.1 (2257/2964) |
| Urgent | 22.6 (670/2964) |
| Emergent | 1.2 (36/2964) |
| Salvage | 0.0 (1/2964) |
| Off-pump | 14.7 (437/2964) |
| Full sternotomy | 99.8 (2948/2955) |
| Vein harvesting technique |  |
| Open | 81.5 (2262/2775) |
| Endoscopic | 14.3 (396/2775) |
| Combined open and endoscopic | 2.9 (81/2775) |
| No-touch | 1.3 (36/2775) |
| Valve surgery ^1^ |  |
| Aortic repair | 0.1 (4/2960) |
| Aortic replacement | 9.7 (286/2960) |
| Mitral repair | 2.5 (73/2960) |
| Mitral replacement | 2.5 (74/2960) |
| Tricuspid repair | 0.9 (27/2960) |
| Complete revascularization | 80.9 (2394/2961) |
| Cumulative cross clamp time (min) | 76 ± 33 (2499) |
| Cumulative bypass time (min) | 108 ± 48 (2511) |

^1^ Patients could have had ≥1 valve procedure.

## Table S4. Details of graft use of the whole cohort including isolated CABG (n=2532) and combined CABG and valve surgery (n=432) patients

|  | **All patients**  **(n=2964)** |
| --- | --- |
| Graft use per patient |  |
| Saphenous vein | 90.6 (2682/2959) |
| LITA | 89.0 (2633/2959) |
| RITA | 15.7 (464/2959) |
| Radial artery | 10.0 (295/2959) |
| Total grafts per patient, mean ± SD | 2.7 ± 0.8 (2959) |
| Vein grafts per patient, mean ± SD | 1.5 ± 0.9 (2959) |
| Vein grafts per patient |  |
| 0 | 9.4 (277/2959) |
| 1 | 44.6 (1319/2959) |
| 2 | 33.7 (996/2959) |
| 3 | 11.1 (327/2959) |
| ≥ 4 | 1.4 (40/2959) |
| Arterial grafts per patient, mean ± SD | 1.1 ± 0.6 (2959) |
| Arterial grafts per patient |  |
| 0 | 9.4 (277/2959) |
| 1 | 69.2 (2049/2959) |
| ≥2 | 21.4 (633/2959) |
| Distal anastomoses, mean ± SD | 2.9 ± 0.9 (2925) |
| Myocardial territory grafted |  |
| Left anterior descending | 92.1 (2696/2928) |
| Circumflex artery | 81.3 (2381/2928) |
| Right coronary artery | 69.0 (2019/2928) |

LITA: left internal thoracic artery; RITA: right internal thoracic artery.

## Table S5. Use of guideline-directed medical therapy of the whole cohort including isolated CABG (n=2532) and combined CABG and valve surgery (n=432) patients

|  | **All patients**  **(n=2964)** | |
| --- | --- | --- |
|  | Discharge | 1 Year |
| Aspirin | 92.6 (2732/2951) | 86.0 (2014/2342) |
| Other anti-platelet agent | 32.3 (953/2951) | 22.8 (534/2342) |
| Any anti-platelet agent | 95.9 (2831/2951) | 92.1 (2158/2342) |
| Vitamin K antagonist | 10.2 (300/2951) | 2.8 (66/2343) |
| DOAC | 4.2 (123/2951) | 6.5 (157/2397) |
| Statin | 87.0 (2567/2950) | 88.8 (2079/2342) |
| Other lipid-lowering agent (non-statin) | 3.6 (105/2950) | 9.3 (217/2342) |
| Beta-blocker | 83.9 (2474/2950) | 85.1 (1994/2344) |
| RAAS-active agent | 62.6 (1848/2950) | 63.7 (1492/2344) |

DOAC: direct oral anticoagulant; RAAS: renin-angiotensin-aldosterone-system.

## Table S6. Patient characteristics of patients who underwent combined CABG and valve surgery

|  | **CABG and valve**  **(n=432)** |
| --- | --- |
| Age, y, mean ± SD | 70.3 ± 8.5 (432) |
| Male | 81.3 (351/432) |
| Caucasian ethnicity | 90.7 (391/431) |
| BMI, kg/m^2^, mean ± SD | 27.9 ± 4.1 (431) |
| Smoking status |  |
| Current smoker | 16.0 (69/431) |
| Ex-smoker | 36.4 (157/431) |
| Never smoked | 47.6 (205/431) |
| Diabetes mellitus | 38.1 (164/430) |
| Insulin treated diabetes mellitus | 11.9 (51/430) |
| Hypertension | 83.0 (357/430) |
| Dyslipidemia | 76.2 (326/428) |
| Renal function ^1^ |  |
| Normal | 33.1 (143/432) |
| Moderately impaired | 48.4 (209/432) |
| Severely impaired | 18.5 (80/432) |
| Cerebrovascular disease ^2^ | 10.5 (45/430) |
| Peripheral vascular disease | 14.1 (60/425) |
| Pulmonary disease | 13.9 (60/432) |
| Pulmonary Hypertension | 27.8 (120/432) |
| Previous myocardial infarction | 26.6 (114/429) |
| Anginal status CCS III and IV | 21.8 (94/432) |
| Atrial fibrillation/ flutter | 12.5 (54/432) |
| Previous PCI | 20.1 (87/432) |
| Previous cardiac surgery | 1.4 (6/432) |
| Active endocarditis | 0.9 (4/432) |
| Extent of coronary artery disease |  |
| 1 vessel disease | 19.4 (83/428) |
| 2 vessel disease | 27.6 (118/428) |
| 3 vessel disease | 53.0 (227/428) |
| Left main disease | 27.1 (115/424) |
| LVEF ≤ 50% | 34.5 (149/432) |
| EuroSCORE II, median (IQR, n) | 3.0 (1.9-4.9, 432) |

^1^ Severe renal impairment: creatine clearance < 50 ml/min; moderate renal impairment: creatine clearance 50-85 ml/min.

^2^ Previous stroke, transient ischemic attack or coma

BMI: body mass index; CCS: Canadian Cardiovascular Society; LVEF: left ventricular ejection fraction; PCI: percutaneous coronary intervention.

## Table S7. Characteristics of the surgical procedure of patients who underwent combined CABG and valve surgery

|  | **CABG and valve**  **(n=432)** |
| --- | --- |
| Status |  |
| Elective | 85.2 (368/432) |
| Urgent | 14.1 (61/432) |
| Emergent | 0.5 (2/432) |
| Salvage | 0.2 (1/432) |
| Off-pump | 0.0 (0/432) |
| Full sternotomy | 100.0 (431/431) |
| Vein harvesting technique |  |
| Open | 86.5 (358/414) |
| Endoscopic | 11.6 (48/414) |
| Combined open and endoscopic | 1.9 (8/414) |
| No-touch | 0.0 (0/414) |
| Valve surgery ^1^ |  |
| Aortic repair | 0.9 (4/432) |
| Aortic replacement | 66.2 (286/432) |
| Mitral repair | 16.9 (73/432) |
| Mitral replacement | 17.1 (74/432) |
| Tricuspid repair | 6.3 (27/432) |
| Complete revascularization | 79.9 (345/432) |
| Cumulative cross clamp time (min) | 112 ± 36 (424) |
| Cumulative bypass time (min) | 150 ± 73 (423) |

^1^ Patients could have had ≥1 valve procedure.

## Table S8. Details of graft use of patients who underwent combined CABG and valve surgery

|  | **CABG and valve**  **(n=432)** |
| --- | --- |
| Graft use per patient |  |
| Saphenous vein | 96.8 (417/431) |
| LITA | 65.9 (284/431) |
| RITA | 3.9 (17/431) |
| Radial artery | 2.3 (10/431) |
| Total grafts per patient, mean ± SD | 2.2 ± 0.9 (431) |
| Vein grafts per patient, mean ± SD | 1.4 ± 0.7 (431) |
| Vein grafts per patient |  |
| 0 | 3.2 (14/431) |
| 1 | 56.3 (247/431) |
| 2 | 31.6 (136/431) |
| 3 | 7.2 (31/431) |
| ≥ 4 | 0.7 (3/431) |
| Arterial grafts per patient, mean ± SD | 0.7 ± 0.6 (431) |
| Arterial grafts per patient |  |
| 0 | 32.9 (142/431) |
| 1 | 62.2 (268/431) |
| ≥2 | 4.9 (21/431) |
| Distal anastomoses, mean ± SD | 2.3 ± 1.0 (418) |
| Myocardial territory grafted |  |
| Left anterior descending | 72.9 (307/421) |
| Circumflex artery | 66.5 (280/421) |
| Right coronary artery | 60.3 (254/421) |

LITA: left internal thoracic artery; RITA: right internal thoracic artery.

## Table S9. Use of guideline-directed medical therapy of patients who underwent combined CABG and valve surgery

|  | **CABG and valve**  **(n=432)** | |
| --- | --- | --- |
|  | Discharge | 1 Year |
| Aspirin | 86.6 (369/426) | 79.8 (257/322) |
| Other anti-platelet agent | 13.8 (59/426) | 10.6 (34/322) |
| Any anti-platelet agent | 89.7 (382/426) | 85.4 (275/322) |
| Vitamin K antagonist | 44.5 (190/427) | 11.1 (36/323) |
| DOAC | 0.7 (3/427) | 6.4 (21/330) |
| Statin | 86.4 (368/426) | 90.0 (289/321) |
| Other lipid-lowering agent (non-statin) | 2.8 (12/426) | 8.4 (27/321) |
| Beta-blocker | 74.9 (319/426) | 77.7 (251/323) |
| RAAS-active agent | 54.9 (234/426) | 62.8 (203/323) |

DOAC: direct oral anticoagulant; RAAS: renin-angiotensin-aldosterone-system.

## Table S10. Hazard ratio for a major adverse cardiac event in an univariable and multivariable model after isolated CABG.

|  | Univariable Models | | Multivariable Models | |
| --- | --- | --- | --- | --- |
| Covariate | Unadjusted Hazard Ratio (95% Confidence Interval) | p-value | Adjusted Hazard Ratio (95% Confidence Interval) | p-value |
| Age (per 10 years) | 1.50 [1.24-1.81] | <0.01 | 1.42 [1.13-1.79] | <0.01 |
| Female sex | 1.19 [0.81-1.75] | 0.39 | 1.08 [0.72-1.62] | 0.71 |
| Operation Status |  | . |  | . |
| Urgent surgery | 1.49 [1.07-2.09] | 0.02 | 0.92 [0.62-1.35] | 0.66 |
| Emergency surgery | 3.49 [1.54-7.96] | <0.01 | 1.53 [0.56-4.16] | 0.41 |
| Diabetes - insulin dependent | 1.51 [1.03-2.23] | 0.04 | 1.25 [0.83-1.90] | 0.29 |
| Renal Impairment |  | . |  | . |
| Moderate renal impairment | 1.55 [1.08-2.21] | 0.02 | 1.11 [0.73-1.69] | 0.63 |
| Severe renal impairment | 2.94 [1.92-4.49] | <0.01 | 1.48 [0.87-2.49] | 0.15 |
| Extracardiac arteriopathy | 2.12 [1.52-2.96] | <0.01 | 1.65 [1.14-2.37] | <0.01 |
| Poor mobility due to any non-cardiac reason | 1.68 [0.93-3.02] | 0.09 | 0.82 [0.42-1.60] | 0.57 |
| Pulmonary disease | 1.44 [0.97-2.15] | 0.07 | 1.19 [0.78-1.81] | 0.43 |
| CCS Class 4 | 1.88 [1.22-2.90] | <0.01 | 1.35 [0.79-2.30] | 0.27 |
| NYHA classification |  | . |  | . |
| Class II | 1.18 [0.78-1.78] | 0.43 | 1.13 [0.74-1.72] | 0.58 |
| Class III | 1.92 [1.24-2.98] | <0.01 | 1.33 [0.83-2.13] | 0.23 |
| Class IV | 3.15 [1.67-5.96] | <0.01 | 1.15 [0.50-2.63] | 0.75 |
| Previous myocardial infarction | 1.54 [1.12-2.11] | <0.01 | 1.30 [0.93-1.83] | 0.13 |
| Previous cardiac surgery | 2.08 [0.77-5.61] | 0.15 | 1.40 [0.44-4.48] | 0.57 |
| Ejection fraction < 50% | 2.08 [1.52-2.83] | <0.01 | 1.79 [1.27-2.53] | <0.01 |
| Pulmonary hypertension | 2.11 [1.38-3.23] | <0.01 | 1.49 [0.94-2.36] | 0.09 |
| Critical pre-operative state | 4.42 [2.64-7.40] | <0.01 | 2.54 [1.37-4.73] | <0.01 |
| Left main disease | 1.65 [1.21-2.26] | <0.01 | 1.53 [1.10-2.12] | 0.01 |
| Type of graft |  | . |  | . |
| Saphenous veins only | 1.72 [0.97-3.04] | 0.06 | 1.08 [0.56-2.09] | 0.82 |
| Arteries only | 1.12 [0.69-1.84] | 0.64 | 1.40 [0.83-2.36] | 0.21 |
| On-pump surgery | 0.97 [0.64-1.45] | 0.87 | 0.99 [0.65-1.51] | 0.95 |

For variables with multiple classes, references were for grafts: combination of arterial and saphenous vein grafts; renal function: normal renal function; NYHA: NYHA class I; operative status: elective status. Severe renal impairment: creatine clearance < 50 ml/min; moderate renal impairment: creatine clearance 50-85 ml/min. Pulmonary disease: COPD (chronic obstructive pulmonary disease; emphysema and asthma). CABG: coronary artery bypass grafting; CCS: Canadian Cardiovascular Society; NYHA: New York Heart Association.

## Table S11. Hazard ratio for a major adverse cardiac and cerebral event for isolated CABG patients in an univariable and multivariable model (n=2532)

|  | Univariable Models | | Multivariable Models | |
| --- | --- | --- | --- | --- |
| Covariate | Unadjusted Hazard Ratio (95% Confidence Interval) | p-value | Adjusted Hazard Ratio (95% Confidence Interval) | p-value |
| Age (per 10 years) | 1.51 (1.27-1.79) | <0.01 | 1.44 (1.17-1.78) | <0.01 |
| Female sex | 1.19 (0.83-1.69) | 0.35 | 1.06 (0.73-1.54) | 0.78 |
| Operation Status |  |  |  |  |
| Urgent surgery | 1.43 (1.05-1.95) | 0.02 | 0.86 (0.60-1.23) | 0.40 |
| Emergency surgery | 2.86 (1.26-6.48) | 0.01 | 1.13 (0.42-3.07) | 0.81 |
| Diabetes - insulin dependent | 1.41 (0.98-2.03) | 0.06 | 1.17 (0.80-1.73) | 0.42 |
| Renal Impairment |  |  |  |  |
| Moderate renal impairment | 1.60 (1.16-2.22) | <0.01 | 1.16 (0.79-1.70) | 0.45 |
| Severe renal impairment | 2.92 (1.97-4.33) | <0.01 | 1.52 (0.94-2.45) | 0.09 |
| Extracardiac arteriopathy | 2.22 (1.64-3.01) | <0.01 | 1.72 (1.23-2.39) | <0.01 |
| Poor mobility due to any non-cardiac reason | 1.65 (0.96-2.84) | 0.07 | 0.80 (0.43-1.49) | 0.48 |
| Pulmonary disease | 1.26 (0.86-1.84) | 0.23 | 1.02 (0.68-1.54) | 0.91 |
| CCS Class 4 | 2.10 (1.43-3.08) | <0.01 | 1.53 (0.94-2.47) | 0.08 |
| NYHA classification |  |  |  |  |
| Class II | 1.22 (0.84-1.77) | 0.31 | 1.16 (0.79-1.71) | 0.44 |
| Class III | 1.70 (1.13-2.56) | 0.01 | 1.19 (0.77-1.85) | 0.43 |
| Class IV | 3.70 (2.13-6.43) | <0.01 | 1.55 (0.77-3.12) | 0.22 |
| Previous myocardial infarction | 1.51 (1.13-2.02) | <0.01 | 1.29 (0.94-1.75) | 0.11 |
| Previous cardiac surgery | 2.20 (0.91-5.36) | 0.08 | 1.15 (0.36-3.66) | 0.81 |
| Ejection fraction < 50% | 2.01 (1.51-2.67) | <0.01 | 1.77 (1.29-2.42) | <0.01 |
| Pulmonary hypertension | 1.89 (1.26-2.84) | <0.01 | 1.35 (0.88-2.09) | 0.17 |
| Critical pre-operative state | 4.19 (2.58-6.81) | <0.01 | 2.61 (1.46-4.67) | <0.01 |
| Left main disease | 1.34 (1.01-1.79) | 0.04 | 1.19 (0.88-1.61) | 0.26 |
| Type of graft |  |  |  |  |
| Saphenous veins only | 1.39 (0.79-2.45) | 0.25 | 0.86 (0.45-1.65) | 0.65 |
| Arteries only | 1.03 (0.64-1.63) | 0.92 | 1.30 (0.80-2.13) | 0.29 |
| On-pump surgery | 1.15 (0.77-1.70) | 0.50 | 1.20 (0.79-1.80) | 0.39 |

For variables with multiple classes, references were: grafts: combination of arterial and saphenous vein grafts; renal function: normal renal function; NYHA: NYHA class I; operative status: elective status. Severe renal impairment: creatine clearance < 50 ml/min; moderate renal impairment: creatine clearance 50-85 ml/min. Pulmonary disease: COPD (chronic obstructive pulmonary disease; emphysema and asthma). CABG: coronary artery bypass grafting; CCS: Canadian Cardiovascular Society; NYHA: New York Heart Association.

## Table S12. Clinical outcomes of the whole cohort including isolated CABG (n=2532) and combined CABG and valve surgery (n=432) patients

|  | **All patients**  **(n=2964)** | |
| --- | --- | --- |
|  | *% (No. of events)* | |
|  | 30 days | 1 year |
| MACE | 4.1 (120) | 7.4 (210) |
| MACCE | 5.2 (153) | 8.6 (247) |
| All-cause death | 2.7 (80) | 5.2 (148) |
| Cardiovascular death | 2.7 (80) | 4.5 (130) |
| Myocardial infarction | 1.6 (46) | 2.2 (63) |
| All repeat revascularization | 1.1 (31) | 2.1 (58) |
| PCI | 0.8 (22) | 1.8 (48) |
| Re-CABG | 0.3 (9) | 0.3 (10) |
| Stroke | 1.7 (50) | 2.3 (65) |

Percentages indicate cumulative event rates by Kaplan Meier estimates.

MACE: major adverse cardiac events; MACCE: major adverse cardiac and cerebrovascular events; PCI, percutaneous coronary intervention; CABG, coronary artery bypass grafting.

## Table S13. Hazard ratio for a major adverse cardiac event for the whole cohort including isolated CABG (n=2532) and combined CABG and valve surgery (n=432) patients in an univariable and multivariable model

|  | **Univariable Models** | | **Multivariable Models** | |
| --- | --- | --- | --- | --- |
| Covariate | Unadjusted Hazard  Ratio (95% Confidence Interval) | p-value | Adjusted Hazard  Ratio (95% Confidence Interval) | p-value |
| Age (per 10 years) | 1.49 (1.27-1.76) | <0.01 | 1.30 (1.06-1.58) | <0.01 |
| Female sex | 1.25 (0.90-1.74) | 0.19 | 1.09 (0.77-1.54) | 0.64 |
| Operation Status |  |  |  |  |
| Urgent surgery | 1.46 (1.08-1.97) | 0.01 | 0.95 (0.67-1.35) | 0.76 |
| Emergency surgery | 3.95 (1.9-8.06) | <0.01 | 2.10 (0.90-4.91) | 0.09 |
| Isolated CABG | 0.52 (0.38-0.72) | <0.01 | 0.49 (0.34-0.72) | <0.0 |
| Diabetes - insulin dependent | 1.42 (1.00-2.01) | 0.06 | 1.19 (0.82-1.73) | 0.35 |
| Renal Impairment |  |  |  |  |
| Moderate renal impairment | 1.69 (1.22-2.34) | <0.01 | 1.30 (0.89-1.89) | 0.17 |
| Severe renal impairment | 3.62 (2.51-5.22) | <0.01 | 2.12 (1.36-3.31) | 0.01 |
| Extracardiac arteriopathy | 1.95 (1.45-2.62) | <0.01 | 1.50 (1.08-2.09) | 0.01 |
| Poor mobility due to any non-cardiac reason | 1.99 (1.23-3.23) | <0.01 | 1.06 (0.61-1.83) | 0.85 |
| Pulmonary disease | 1.34 (0.94-1.92) | 0.10 | 1.16 (0.80-1.70) | 0.43 |
| CCS Class 4 | 1.99 (1.36-2.91) | <0.01 | 1.62 (1.02-2.56) | 0.04 |
| NYHA classification |  |  |  |  |
| Class II | 1.33 (0.91-1.95) | 0.14 | 1.19 (0.80-1.77) | 0.39 |
| Class III | 2.16 (1.46-3.21) | 0.01 | 1.39 (0.91-2.13) | 0.13 |
| Class IV | 3.32 (1.85-5.96) | <0.01 | 0.96 (0.45-2.02) | 0.91 |
| Previous myocardial infarction | 1.47 (1.11-1.93) | <0.01 | 1.34 (0.99-1.81) | 0.06 |
| Previous cardiac surgery | 2.39 (1.06-5.39) | 0.043 | 2.00 (0.81-4.91) | 0.13 |
| Ejection fraction < 50% | 1.84 (1.40-2.42) | <0.01 | 1.59 (1.18-2.15) | <0.01 |
| Pulmonary hypertension | 1.74 (1.22-2.48) | <0.01 | 1.08 (0.73-1.59) | 0.72 |
| Critical pre-operative state | 4.09 (2.58-6.49) | <0.01 | 2.41 (1.40-4.14) | <0.01 |
| Left main disease | 1.52 (1.16-2.00) | <0.01 | 1.49 (1.11-1.99) | <0.01 |
| Type of graft |  |  |  |  |
| Saphenous veins only | 1.70 (1.15-2.51) | <0.01 | 1.11 (0.71-1.74) | 0.65 |
| Arteries only | 1.01 (0.63-1.63) | 0.95 | 1.35 (0.81-2.23) | 0.25 |
| On-pump surgery | 1.08 (0.73-1.60) | 0.70 | 0.94 (0.62-1.43) | 0.78 |

For variables with multiple classes, references were for grafts: combination of arterial and saphenous vein grafts; renal function: normal renal function; NYHA: NYHA class I; operative status: elective status. Severe renal impairment: creatine clearance < 50 ml/min; moderate renal impairment: creatine clearance 50-85 ml/min. Pulmonary disease: COPD (chronic obstructive pulmonary disease; emphysema and asthma). CABG: coronary artery bypass grafting; CCS: Canadian Cardiovascular Society; NYHA: New York Heart Association.

## Table S14. Hazard ratio for a major adverse cardiac and cerebral event for the whole cohort including isolated CABG (n=2532) and combined CABG and valve surgery (n=432) patients in an univariable and multivariable model

|  | **Univariable Models** | | **Multivariable Models** | |
| --- | --- | --- | --- | --- |
| Covariate | Unadjusted Hazard Ratio (95% Confidence Interval) | p-value | Adjusted Hazard Ratio (95% Confidence Interval) | p-value |
| Age (per 10 years) | 1.47 (1.26-1.71) | <0.01 | 1.30 (1.09-1.55) | <0.01 |
| Female sex | 1.33 (0.98-1.79) | 0.07 | 1.16 (0.84-1.60) | 0.36 |
| Operation Status |  |  |  |  |
| Urgent surgery | 1.46 (1.11-1.93) | <0.01 | 0.93 (0.67-1.29) | 0.67 |
| Emergency surgery | 3.29 (1.62-6.69) | 0.01 | 1.65 (0.71-3.82) | 0.24 |
| Isolated CABG | 0.54 (0.40-0.73) | <0.01 | 0.52 (0.37-0.73) | <0.01 |
| Diabetes - insulin dependent | 1.31 (0.94-1.82) | 0.11 | 1.08 (0.76-1.53) | 0.68 |
| Renal Impairment |  |  |  |  |
| Moderate renal impairment | 1.67 (1.24-2.24) | <0.01 | 1.27 (0.90-1.79) | 0.17 |
| Severe renal impairment | 3.44 (2.46-4.83) | <0.01 | 2.02 (1.34-3.05) | <0.01 |
| Extracardiac arteriopathy | 2.08 (1.59-2.73) | <0.01 | 1.65 (1.22-2.21) | 0.01 |
| Poor mobility due to any non-cardiac reason | 1.88 (1.19-2.97) | <0.01 | 0.99 (0.59-1.67) | 0.97 |
| Pulmonary disease | 1.17 (0.83-1.65) | 0.36 | 1.01 (0.70-1.45) | 0.98 |
| CCS Class 4 | 2.17 (1.54-3.06) | <0.01 | 1.78 (1.17-2.69) | <0.01 |
| NYHA classification |  |  |  |  |
| Class II | 1.36 (0.96-1.93) | 0.08 | 1.22 (0.85-1.75) | 0.27 |
| Class III | 2.01 (1.39-2.90) | <0.01 | 1.31 (0.88-1.94) | 0.19 |
| Class IV | 3.75 (2.23-6.30) | <0.01 | 1.22 (0.64-2.32) | 0.55 |
| Previous myocardial infarction | 1.52 (1.18-1.95) | <0.01 | 1.36 (1.03-1.80) | 0.03 |
| Previous cardiac surgery | 2.40 (1.13-5.09) | 0.02 | 1.63 (0.66-4.00) | 0.29 |
| Ejection fraction < 50% | 1.84 (1.44-2.37) | <0.01 | 1.62 (1.23-2.13) | <0.01 |
| Pulmonary hypertension | 1.62 (1.16-2.27) | <0.01 | 1.02 (0.70-1.47) | 0.93 |
| Critical pre-operative state | 3.83 (2.47-5.93) | <0.01 | 2.32 (1.39-3.87) | <0.01 |
| Left main disease | 1.27 (0.98-1.63) | 0.07 | 1.19 (0.91-1.56) | 0.21 |
| Type of graft |  |  |  |  |
| Saphenous veins only | 1.50 (1.03-2.18) | 0.03 | 0.99 (0.64-1.52) | 0.96 |
| Arteries only | 0.93 (0.59-1.46) | 0.75 | 1.24 (0.77-2.00) | 0.37 |
| On-pump surgery | 1.26 (0.86-1.85) | 0.23 | 1.12 (0.75-1.68) | 0.57 |

For variables with multiple classes, references were for grafts: combination of arterial and saphenous vein grafts; renal function: normal renal function; NYHA: NYHA class I; operative status: elective status. Severe renal impairment: creatine clearance < 50 ml/min; moderate renal impairment: creatine clearance 50-85 ml/min. Pulmonary disease: COPD (chronic obstructive pulmonary disease; emphysema and asthma). CABG: coronary artery bypass grafting; CCS: Canadian Cardiovascular Society; NYHA: New York Heart Association.

## Table S15. Clinical outcomes of combined CABG and valve surgery patients

|  | **CABG and valve**  **(n=432)** | |
| --- | --- | --- |
|  | *% (No. of events)* | |
|  | 30 days | 1 year |
| MACE | 7.5 (32) | 12.0 (50) |
| MACCE | 8.9 (38) | 13.6 (57) |
| All-cause death | 5.4 (23) | 9.9 (41) |
| Cardiovascular death | 5.4 (23) | 9.1 (38) |
| Myocardial infarction | 3.1 (13) | 3.3 (14) |
| All repeat revascularization | 0.7 (3) | 1.3 (5) |
| PCI | 0.2 (1) | 0.8 (3) |
| Re-CABG | 0.5 (2) | 0.5 (2) |
| Stroke | 3.1 (13) | 4.6 (19) |

Percentages indicate cumulative event rates by Kaplan Meier estimates.

MACE: major adverse cardiac events; MACCE: major adverse cardiac and cerebrovascular events; PCI, percutaneous coronary intervention; CABG, coronary artery bypass grafting.

## Table S16. Hazard ratio for a major adverse cardiac event for patients with combined CABG and valve surgery (n=432) in an univariable and multivariable model

|  | **Univariable Models** | | **Multivariable Models** | |
| --- | --- | --- | --- | --- |
| Covariate | Unadjusted Hazard Ratio (95% Confidence Interval) | p-value | Adjusted Hazard Ratio(95% Confidence Interval) | p-value |
| Age (per 10 years) | 1.27 (0.89-1.80) | 0.19 | 0.97 (0.64-1.46) | 0.87 |
| Female sex | 1.44 (0.75-2.75) | 0.27 | 1.01 (0.47-2.18) | 0.98 |
| Operation Status |  |  |  |  |
| Urgent surgery | 1.84 (0.94-3.60) | 0.08 | 0.89 (0.38-2.11) | 0.80 |
| Emergency surgery | 17.72 (4.18-75.13) | <0.01 | 4.14 (0.43-40.13) | 0.22 |
| Diabetes mellitus - insulin dependent | 1.22 (0.55-2.72) | 0.62 | 1.02 (0.43-2.40) | 0.96 |
| Renal Impairment |  |  |  |  |
| Moderate renal impairment | 2.31 (0.99-5.39) | 0.05 | 2.59 (1.04-6.49) | 0.04 |
| Severe renal impairment | 5.87 (2.48-13.88) | <0.01 | 6.51 (2.41-17.61) | <0.001 |
| Extracardiac arteriopathy | 1.52 (0.78-2.97) | 0.22 | 1.21 (0.55-2.66) | 0.63 |
| Poor mobility due to any non-cardiac reason | 3.45 (1.47-8.10) | <0.01 | 1.68 (0.48-5.93) | 0.42 |
| Pulmonary disease | 1.05 (0.47-2.33) | 0.91 | 1.22 (0.48-3.11) | 0.67 |
| CCS Class 4 | 3.37 (1.51-7.49) | <0.01 | 4.08 (1.55-10.77) | <0.01 |
| NYHA classification |  |  |  |  |
| Class II | 2.01 (0.60-6.74) | 0.26 | 1.15 (0.33-4.03) | 0.83 |
| Class III | 2.58 (0.78-8.59) | 0.12 | 1.70 (0.49-5.91) | 0.41 |
| Class IV | 4.01 (0.81-19.86) | 0.09 | 0.85 (0.12-6.01) | 0.87 |
| Previous myocardial infarction | 1.79 (1.01-3.16) | 0.05 | 1.43 (0.69-2.95) | 0.34 |
| Previous cardiac surgery | 3.42 (0.83-14.09) | 0.09 | 4.11 (0.85-19.93) | 0.08 |
| Ejection fraction < 50% | 1.18 (0.67-2.09) | 0.56 | 0.97 (0.49-1.94) | 0.94 |
| Pulmonary hypertension | 0.80 (0.42-1.52) | 0.49 | 0.62 (0.30-1.29) | 0.20 |
| Critical pre-operative state | 2.97 (1.07-8.26) | 0.04 | 2.53 (0.62-10.34) | 0.20 |
| Left main disease | 1.52 (0.84-2.74) | 0.17 | 1.72 (0.89-3.35) | 0.11 |
| Type of graft |  |  |  |  |
| Saphenous veins only | 1.01 (0.56-1.82) | 0.98 | 1.07 (0.55-2.05) | 0.85 |
| Arteries only | 0.60 (0.082-4.42) | 0.62 | 0.80 (0.10-6.33) | 0.83 |
| On-pump surgery | 0.39 (0.05-2.84) | 0.35 | 0.20 (0.02-1.67) | 0.14 |

For variables with multiple classes, references were for grafts: combination of arterial and saphenous vein grafts; renal function: normal renal function; NYHA: NYHA class I; operative status: elective status. Severe renal impairment: creatine clearance < 50 ml/min; moderate renal impairment: creatine clearance 50-85 ml/min. Pulmonary disease: COPD (chronic obstructive pulmonary disease; emphysema and asthma). CABG: coronary artery bypass grafting; CCS: Canadian Cardiovascular Society; NYHA: New York Heart Association.

## Table S17. Hazard ratio for a major adverse cardiac and cerebral cardiac event for patients with combined CABG and valve surgery (n=432) in an univariable and multivariable model

|  | **Univariable Models** | | **Multivariable Models** | |
| --- | --- | --- | --- | --- |
| Covariate | Unadjusted Hazard Ratio (95% Confidence Interval) | p-value | Adjusted Hazard Ratio(95% Confidence Interval) | p-value |
| Age (per 10 years) | 1.17 (0.85-1.61) | 0.34 | 0.96 (0.66-1.39) | 0.81 |
| Female sex | 1.81 (1.02-3.23) | 0.04 | 1.56 (0.79-3.06) | 0.20 |
| Operation Status |  |  |  |  |
| Urgent surgery | 2.19 (1.20-4.02) | 0.01 | 1.04 (0.48-2.25) | 0.92 |
| Emergency surgery | 14.50 (3.45-60.82) | <0.01 | 3.35 (0.33-34.3) | 0.31 |
| Diabetes mellitus - insulin dependent | 1.05 (0.48-2.31) | 0.91 | 0.84 (0.36-1.93) | 0.68 |
| Renal Impairment |  |  |  |  |
| Moderate renal impairment | 1.75 (0.84-3.65) | 0.13 | 1.87 (0.84-4.19) | 0.13 |
| Severe renal impairment | 4.51 (2.13-9.52) | <0.01 | 4.17 (1.72-10.11) | <0.01 |
| Extracardiac arteriopathy | 1.76 (0.96-3.22) | 0.07 | 1.44 (0.72-2.91) | 0.30 |
| Poor mobility due to any non-cardiac reason | 3.01 (1.29-7.01) | 0.01 | 1.25 (0.36-4.35) | 0.72 |
| Pulmonary disease | 0.90 (0.41-1.99) | 0.80 | 1.08 (0.44-2.68) | 0.86 |
| CCS Class 4 | 3.48 (1.65-7.35) | 0.01 | 3.80 (1.54-9.38) | <0.01 |
| NYHA classification |  |  |  |  |
| Class II | 2.32 (0.70-7.70) | 0.17 | 1.43 (0.41-4.92) | 0.57 |
| Class III | 3.06 (0.93-10.10) | 0.07 | 2.19 (0.64-7.49) | 0.21 |
| Class IV | 4.15 (0.84-20.55) | 0.08 | 0.90 (0.13-6.12) | 0.91 |
| Previous myocardial infarction | 2.16 (1.27-3.65) | <0.01 | 1.72 (0.89-3.34) | 0.11 |
| Previous cardiac surgery | 3.13 (0.76-12.85) | 0.11 | 4.36 (0.90-21.25) | 0.07 |
| Ejection fraction < 50% | 1.32 (0.78-2.24) | 0.31 | 1.10 (0.59-2.07) | 0.76 |
| Pulmonary hypertension | 0.82 (0.45-1.51) | 0.53 | 0.60 (0.31-1.19) | 0.14 |
| Critical pre-operative state | 2.62 (0.95-7.23) | 0.06 | 2.37 (0.59-9.63) | 0.23 |
| Left main disease | 1.34 (0.76-2.35) | 0.31 | 1.55 (0.83-2.90) | 0.17 |
| Type of graft |  |  |  |  |
| Saphenous veins only | 0.98 (0.56-1.70) | 0.94 | 1.10 (0.59-2.04) | 0.77 |
| Arteries only | 0.52 (0.07-3.80) | 0.52 | 0.66 (0.09-5.14) | 0.69 |
| On-pump surgery | 0.46 (0.06-3.33) | 0.44 | 0.25 (0.03-2.07) | 0.20 |

For variables with multiple classes, references were for grafts: combination of arterial and saphenous vein grafts; renal function: normal renal function; NYHA: NYHA class I; operative status: elective status. Severe renal impairment: creatine clearance < 50 ml/min; moderate renal impairment: creatine clearance 50-85 ml/min. Pulmonary disease: COPD (chronic obstructive pulmonary disease; emphysema and asthma). CABG: coronary artery bypass grafting; CCS: Canadian Cardiovascular Society; NYHA: New York Heart Association.

## Supplementary Figures

## Figure S1. Cumulative incidence of adverse events for the whole cohort including isolated CABG (n=2532) and combined CABG and valve surgery (n=432) patients at 1 year

**
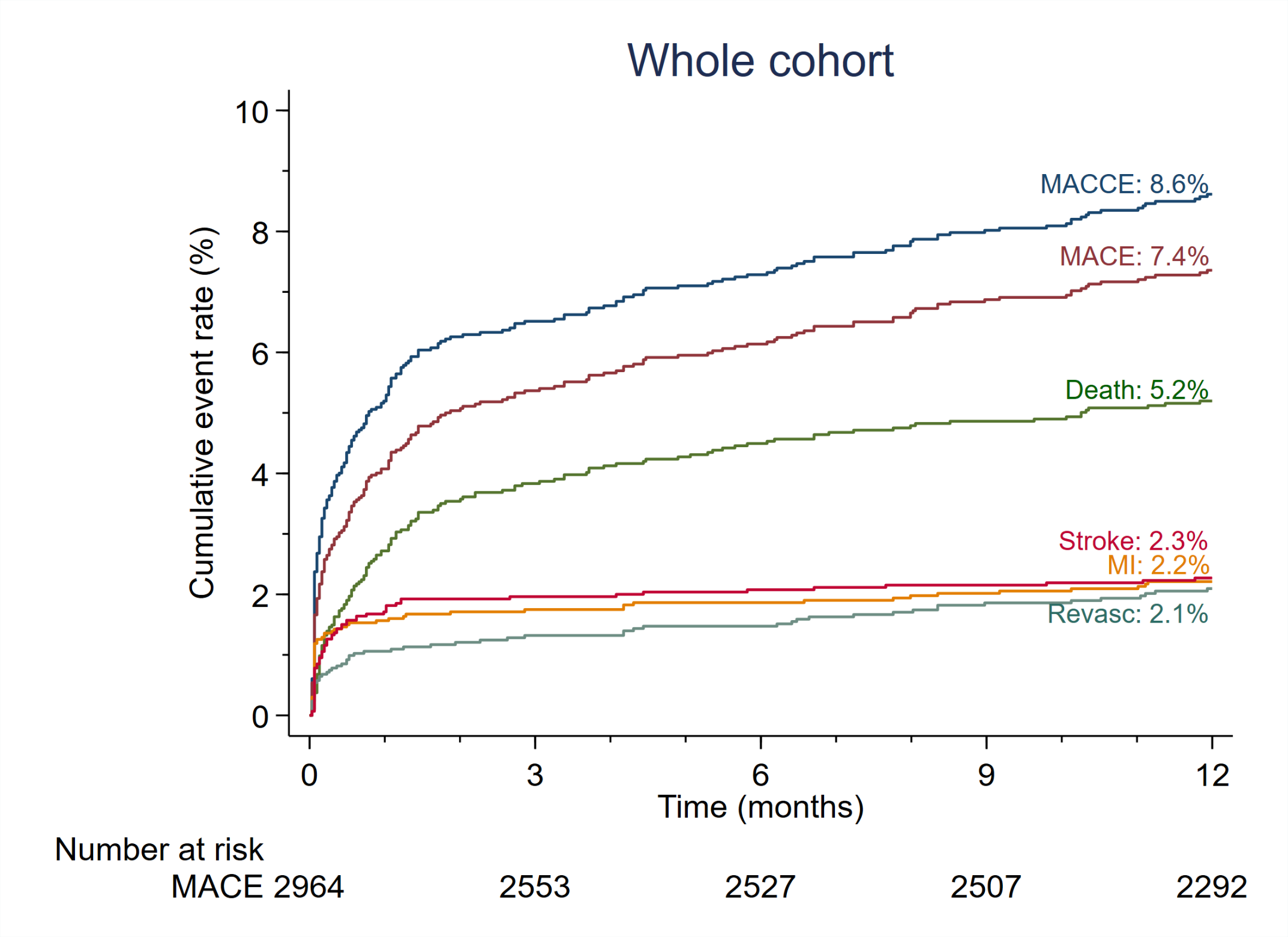
**

## Figure S2. Cumulative incidence of adverse events after combined CABG and valve surgery at 1 year


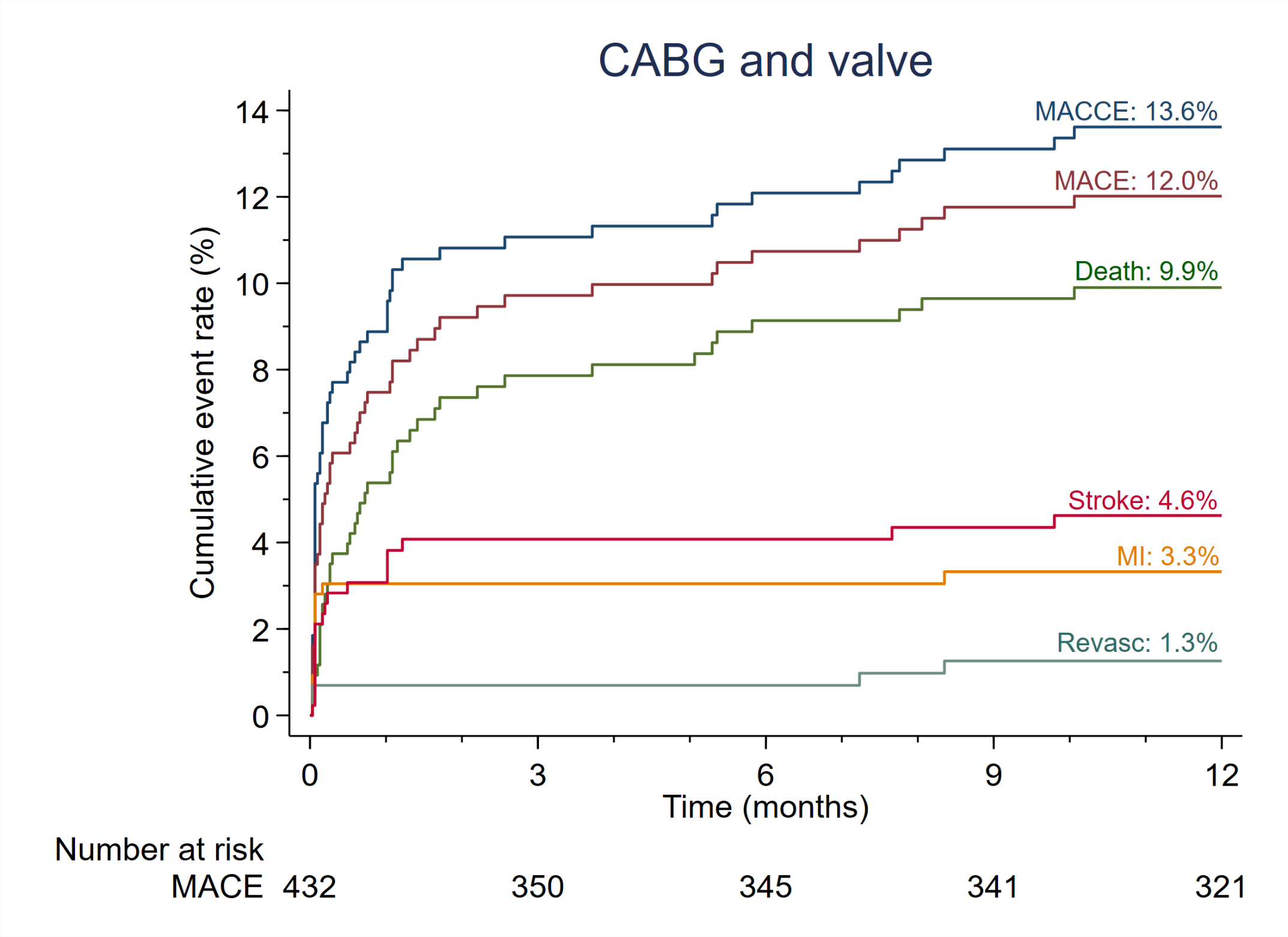


## Figure S3. Median and IQR of the EQ-5D index values for the whole cohort including isolated CABG (n=2532) and combined CABG and valve surgery (n=432) patients at baseline and 1 year


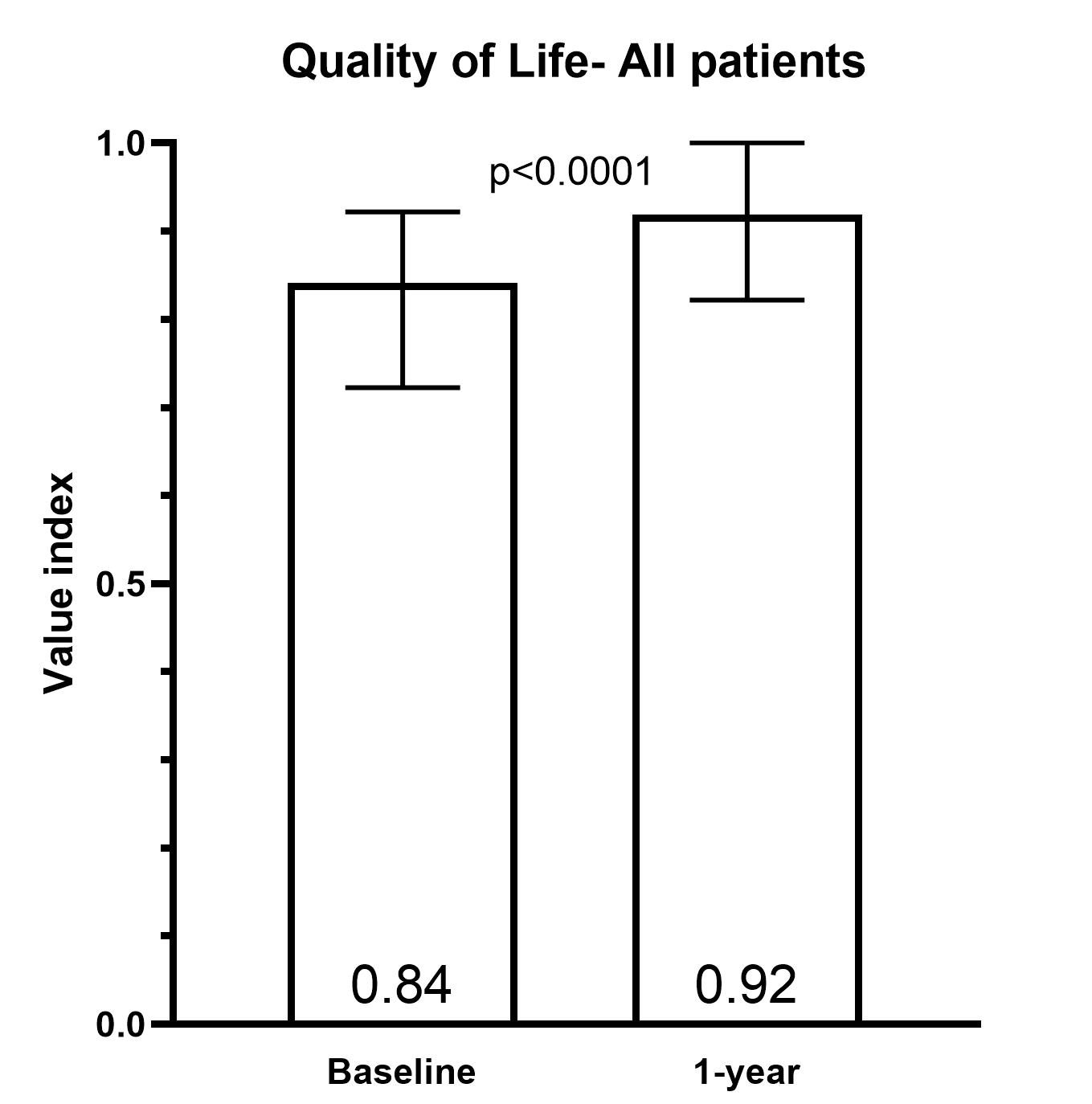


## Figure S4. The five dimensions of the EQ-5D for the whole cohort including isolated CABG (n=2532) and combined CABG and valve surgery (n=432) patients at baseline and 1 year


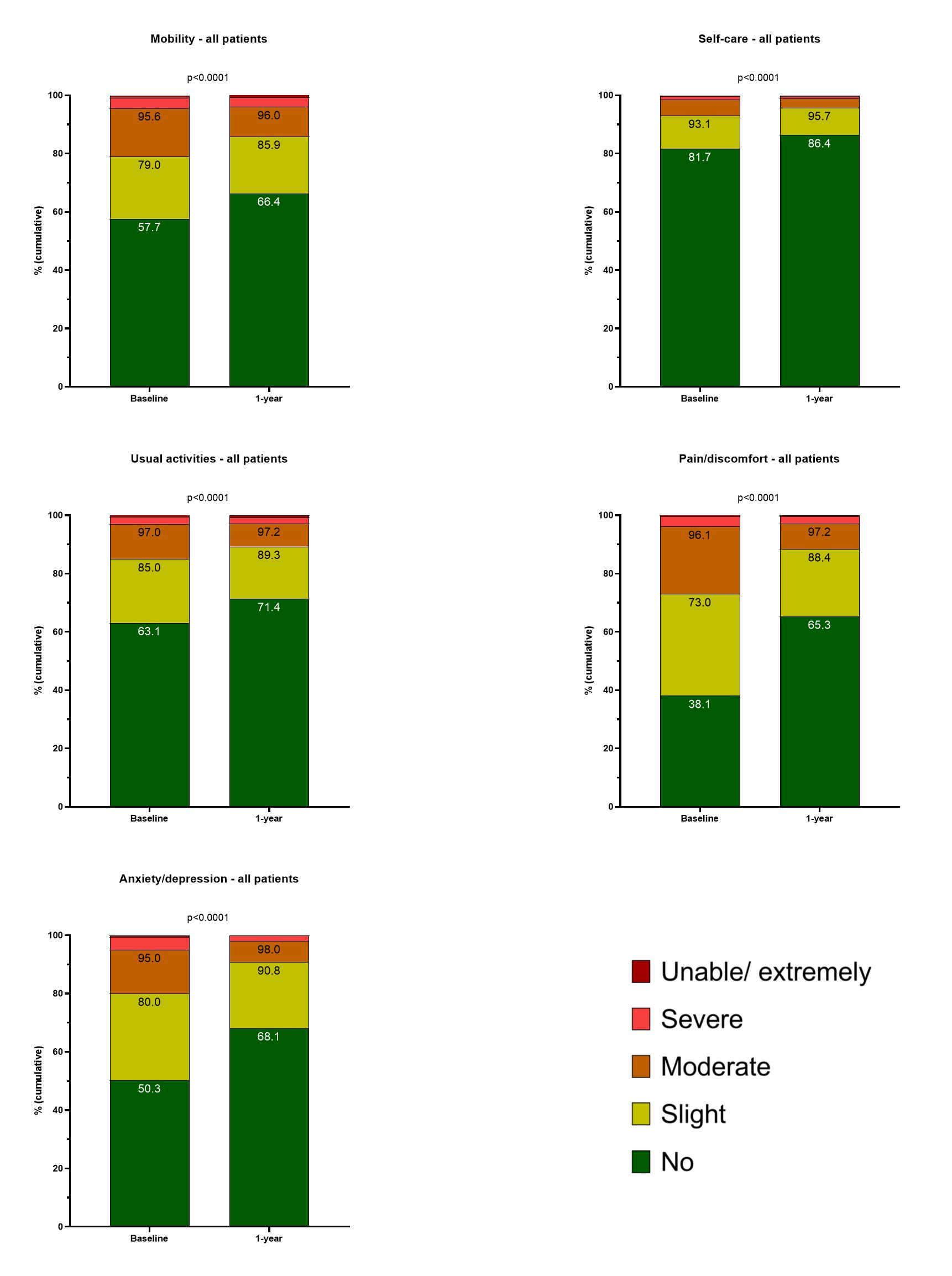


## Figure S5. Median and IQR of the EQ-5D index values for the combined CABG and valve patients (n=432) at baseline and 1 year


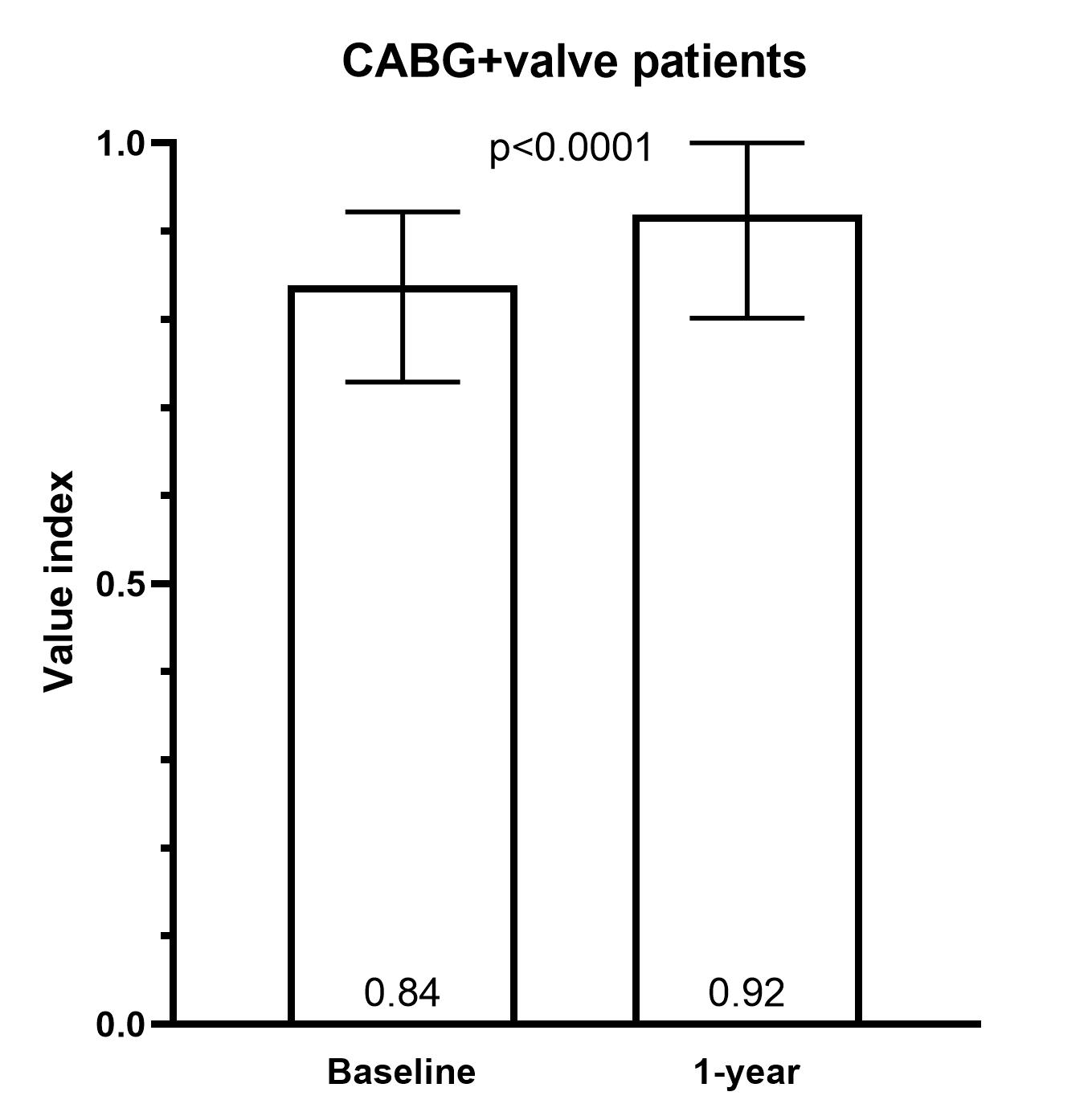


## Figure S6. The five dimensions of the EQ-5D for the combined CABG and valve patients (n=432) at baseline and 1 year


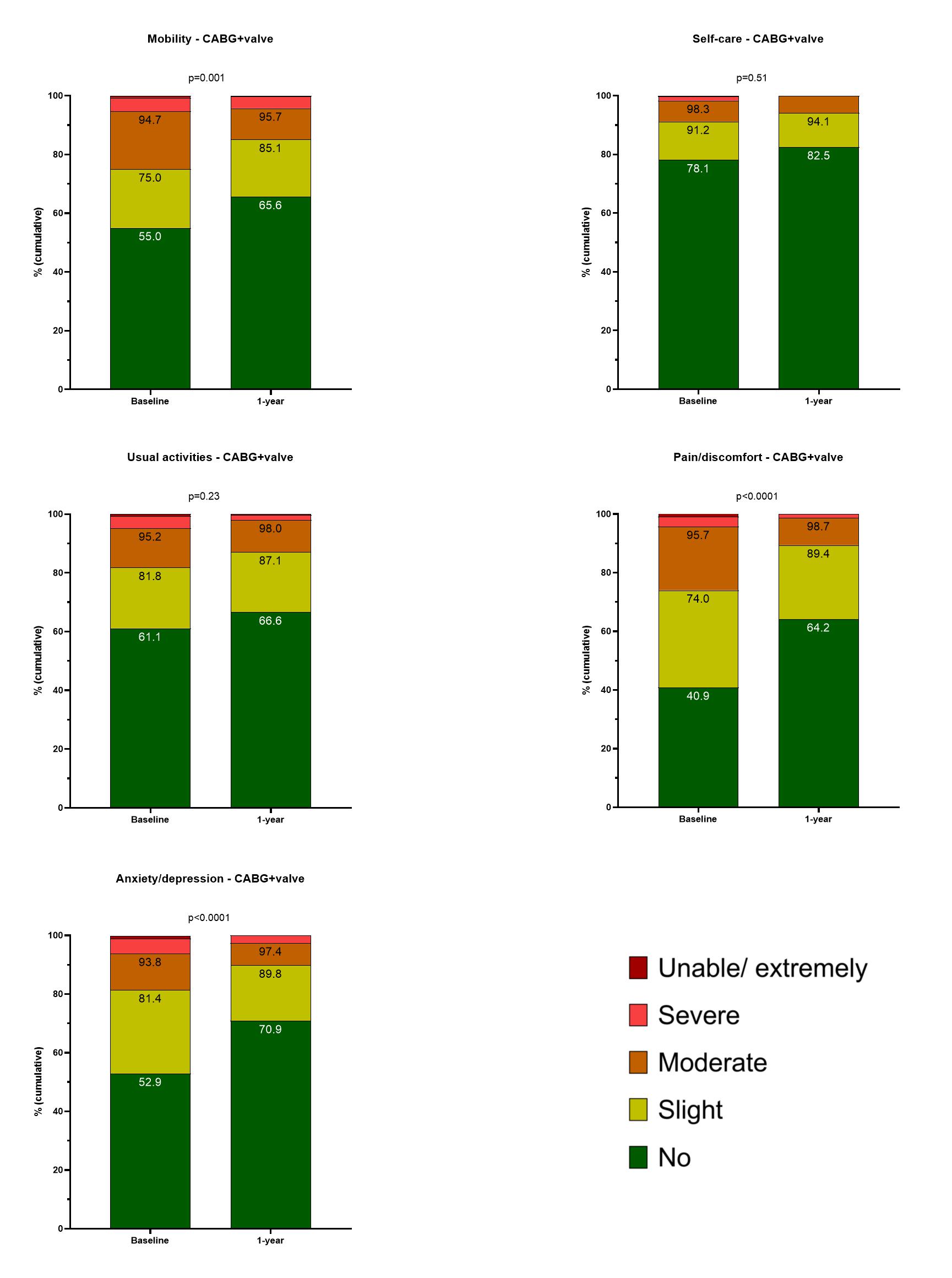

Supplement: Supplementary file 1 [file js9-109-0707-s001.docx]
